# Drivers and Mechanisms of Ecosystem Multifunctionality in Secondary Tropical Forests

**DOI:** 10.1007/s10021-026-01047-1

**Published:** 2026-02-17

**Authors:** Tomonari Matsuo, Lucy Amissah, Masha T. van der Sande, Fons van der Plas, Jazz Kok, Salim Mohammed Abdul, Lucas Chojnacki, Tijs Kuzee, Lhouyangdar Khulpu, Lourens Poorter

**Affiliations:** 1 Forest Ecology and Forest Management Group, Wageningen University, Wageningen, The Netherlands; 2 CSIR-Forestry Research Institute of Ghana, Kumasi, Ghana; 3 CSIR College of Science and Technology, P.O. Box M 32, Accra, Ghana; 4 Plant Ecology and Nature Conservation Group, Wageningen University, Wageningen, The Netherlands

**Keywords:** biodiversity, biogeochemical cycles, ecosystem multifunctionality, forest structure, functional trait composition, macroclimate, secondary tropical forests, soil properties

## Abstract

Tropical forests provide vital ecosystem functions and services, yet global change is intensifying disturbance regimes and expanding the extent of young secondary forests. The question is to what extent these young forests recover ecosystem multifunctionality and under which conditions recovery proceeds faster. Here, we analyze the drivers and mechanisms that shape a comprehensive set of 16 ecosystem functions related to carbon, water, and nutrient cycling. We established plots in 36 young secondary forest stands (2.3–3.6 years since agricultural abandonment) in dry and wet regions in Ghana. In each plot, we measured eight forest attributes related to structure, diversity, and functional composition, six soil physical and chemical properties, and quantified 16 ecosystem functions to test how environmental conditions and forest attributes shape ecosystem functioning using structural equation modeling. Climatic wetness and soil conditions most strongly influenced ecosystem functions (9 functions each), followed by structure (8), diversity (5), and functional composition (4). The relative importance of these drivers and mechanisms varied across cycles: The carbon cycle was most influenced by forest attributes, whereas the water and nutrient cycles were primarily shaped by environmental conditions, with forest structure additionally shaping nutrient cycling. Hence, in early successional ecosystems, ecosystem multifunctionality is more strongly shaped by environmental conditions and vegetation quantity than by biodiversity, and different conditions are needed for the recovery of different cycles. When previous land use intensity has been low, forest recovery can proceed fast, and natural regeneration offers a scalable, low-cost opportunity for restoring multifunctionality, especially in wet tropical forests.

**Supplementary Information:**

The online version contains supplementary material available at 10.1007/s10021-026-01047-1.

## Highlights

- Abiotic conditions strongly shape multifunctionality across biogeochemical cycles
- Vegetation quantity outweighs vegetation quality in shaping multifunctionality
- Water and nutrient cycling are primarily shaped by abiotic conditions
- Carbon cycling is primarily shaped by forest structure and species diversity
- Natural regeneration provides a low-cost opportunity to restore multifunctionality

## Introduction

Tropical forests are crucial for carbon, water, and nutrient cycling, thereby contributing to climate change mitigation, climate regulation, and soil health from local to global scales (IPBES 2019; Balvanera and others 2021). However, global climate and land use changes are driving forest loss and shift in environmental conditions and vegetation properties, which in turn reduce ecosystem functioning (Ratcliffe and others 2017; IPBES 2019; van der Plas 2019). Yet, tropical forests have the potential to regenerate naturally and recover ecosystem functioning through secondary succession (Odum 1969; Poorter and others 2024). Currently, secondary forests account for over half of the tropical forest area, with more than half of these forests being less than 10 years old (FAO 2020; Chazdon and others 2025), highlighting their growing role in the restoration of ecosystem functioning. Nevertheless, most existing studies (1) were conducted in mature, species-rich, and well-developed ecosystems, which may function fundamentally differently from young, species-poor, and developing ecosystems (Lohbeck and others 2015; Matsuo and others 2025a); (2) focused on carbon and/or nutrient cycling, while largely overlooking water cycling, despite its central role in sustaining life; and 3) assessed a single or few functions per biogeochemical cycle rather than multiple functions (Finegan and others 2015; Poorter and others 2015). To address these knowledge gaps, this study aims to analyze how abiotic and biotic drivers shape ecosystem multifunctionality across carbon, water, and nutrient cycling in young secondary tropical forests.

Several mechanisms have been proposed to explain how different drivers shape ecosystem functioning. First, many studies have focused on the (1) *Biodiversity-ecosystem functioning (BEF) hypothesis.* This states that diverse forests can support higher levels of ecosystem functioning than species-poor ones through enhancing resource capture and use efficiency (niche complementarity hypothesis; Loreau 1998; Tilman 1999), increasing the chance to include a highly functioning species (sampling hypothesis; Huston 1979), reducing the prevalence of species-specific pathogens (pathogen dilution hypothesis; Schnitzer and others 2011), and increasing microbial abundance and activities by providing diverse energy sources (energy diversity hypothesis; Enriquez and others 1993). While the BEF hypothesis has been intensively studied, relationships between biodiversity and ecosystem functioning tend to be mixed, and sometimes, other drivers and mechanisms related to functional composition, structure, and environmental conditions are more important in driving ecosystem functioning (van der Plas 2019). (2) *Mass-ratio hypothesis*: dominant species strongly shape functional characteristics of the community (that is functional composition), and thus ecosystem functioning (Grime 1998). For instance, increased dominance of acquisitive species with high leaf nutrient concentrations increases carbon cycling through their inherently fast growth (Finegan 2015) and nutrient cycling through producing a greater quantity and quality of litter (Lohbeck and others 2015). (3) *Vegetation quantity hypothesis*: a structurally developed forest stand possesses a large photosynthetically active leaf area, which enhances carbon sequestration (Lohbeck and others 2015; Matsuo and others 2025a). It also improves soil water-holding capacity as root growth improves soil porosity and vegetation cover reduces soil evaporation (Lebrija-Trejos and others 2010; Falk and others 2024). Additionally, high productivity leads to increased litterfall, contributing to the buildup of soil organic carbon and nutrients (Feng and others 2019); (4) *Environmental drivers hypothesis*: favorable abiotic conditions (wetter and more fertile soils) facilitate tree performance and thus carbon sequestration (Poorter and others 2017; van der Sande and others 2017). Additionally, these conditions increase soil microbial abundance and activities, resulting in faster litter decomposition and mineralization rates (Camenzind and others 2018). Alternatively, very wet conditions may reduce forest productivity by limiting annual radiation (Poorter and others 2015) and may slow litter decomposition and mineralization by constraining microbial activity under anaerobic soil conditions or through nutrient leaching. Therefore, a comprehensive assessment of multiple ecosystem functions across the three major biogeochemical cycles is needed to reveal how the underlying drivers and mechanisms vary within and across cycles.

Most knowledge on biodiversity–ecosystem functioning and the three other mechanisms comes from mature ecosystems, where vegetation quality (that is plant diversity and community composition) plays a central role in driving ecosystem functioning due to high taxonomic and functional diversity (Finegan and others 2015; Poorter and others 2015). In young regenerating forests, vegetation quality may be more important, as diversity is low in early successional systems, and each additional species can provide complementary functions (Cardinale 2011). Alternatively, they may be less important because the dominance of a few functionally similar pioneer species and a relatively high supply of resources (for example light) may reduce the benefits of diversity for efficient resource acquisition and use (Lohbeck and others 2015, 2016). In contrast, vegetation quantity may be more important for ecosystem functioning in these early successional stages, as vegetation is still building up. Therefore, assessing the drivers and mechanisms of multiple ecosystem functions in young tropical forests is essential to test whether and how these mechanisms apply during early succession, and to design effective forest restoration strategies.

**Figure 1:**
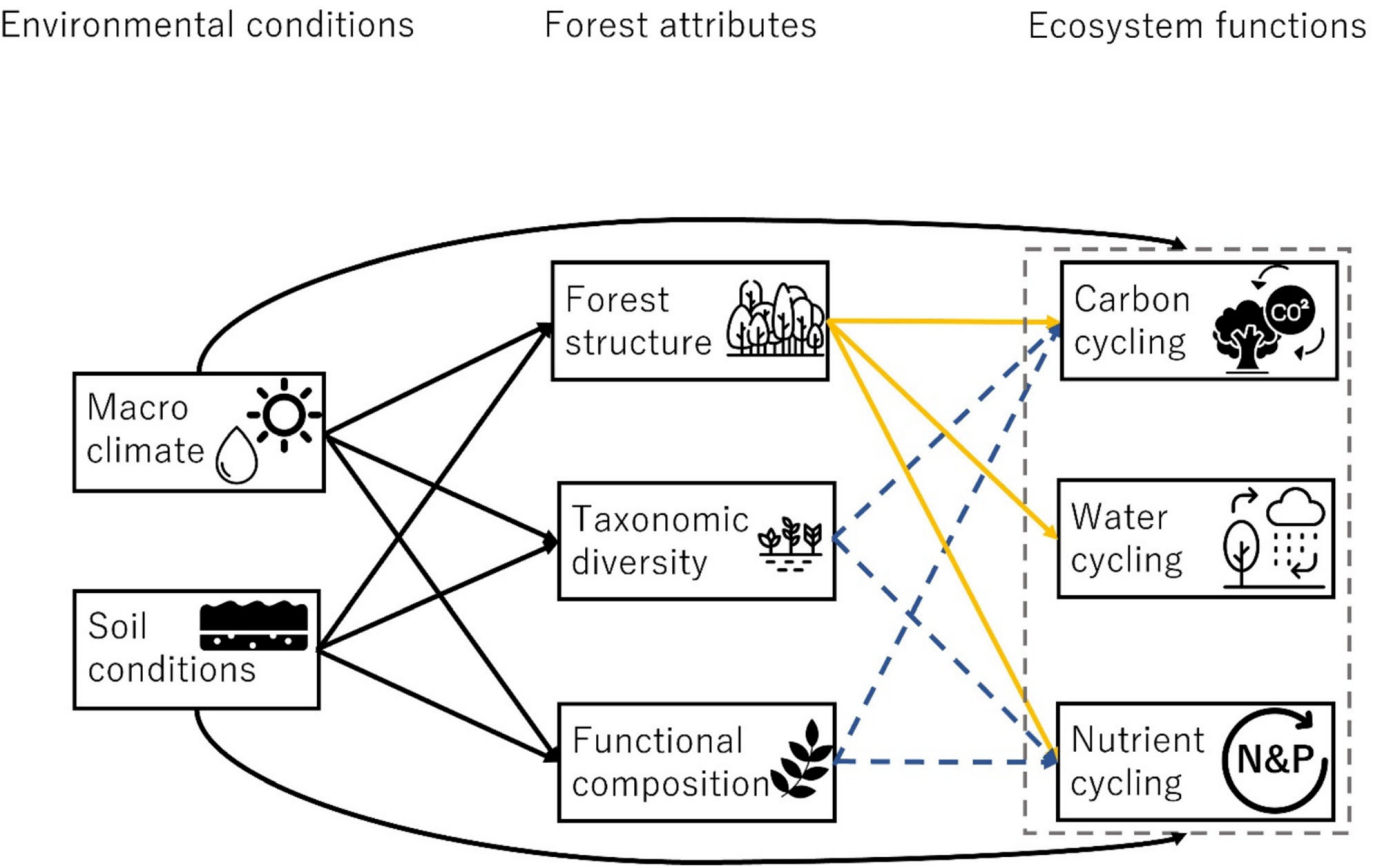
Conceptual model visualizing the hierarchical relationships among environmental conditions (macroclimate and soil conditions), forest attributes (forest structure, taxonomic diversity, and functional composition), and ecosystem functions (carbon-, water-, and nutrient cycling), in both young and old forests. Although all mechanisms (that is pairwise relationships) can play a role in young and old forests, some relationships are expected to be stronger in young forests (orange solid arrows) and others are expected to be stronger in mature forests (dark blue dashed lines) (source icons: Flaticon.com).

This study addresses the question of how environmental conditions and forest attributes determine ecosystem functioning in carbon-, water-, and nutrient cycling in young, secondary tropical forests (Figure 1). We provide a comprehensive perspective by assessing 4–6 functions per cycle (Table 1). We test the overall hypothesis that ecosystem functions are most strongly driven by environmental conditions and forest structure as they fuel productivity and, hence, carbon, water, and nutrient cycling; moderately driven by functional composition as it determines whether forests cycle resources more ‘quickly’ or ‘slowly’; and least by taxonomic diversity, given the high functional similarity among dominant pioneer species in early succession (van der Sande and others 2024). Additionally, we hypothesize that three biogeochemical cycles are shaped by different drivers and mechanisms. Specifically:

1. Carbon functions will increase with a) more favorable environmental conditions that increase tree performance, b) greater vegetation quantity associated with a larger photosynthetic stand leaf area, c) increased diversity through niche complementarity, and d) increased dominance of acquisitive species that have inherently fast growth.
2. Water functions will increase with a) more favorable macroclimatic conditions through increased water input, b) fine-textured and less compacted soil that improves water infiltration and water-holding capacity, and c) greater vegetation quantity that improves soil porosity.
3. Nutrient functions will increase with a) more favorable environmental conditions that increase microbial abundance and activities, b) greater vegetation quantity that increases litter production, c) increased diversity by providing diverse energy sources to microorganisms, and d) increased dominance of acquisitive species that increase the quantity and quality of litter production.

**Table 1:** Overview of the 16 Ecosystem Functions Belonging to Three Biogeochemical Cycles (Carbon, Nutrients, and Water). The Ecosystem Fluxes (Influx, Internal Stock or Flow, or Efflux), Units, and Descriptions are Indicated

Biogeochemical cycling	Fluxes within the ecosystem	Ecosystem function	Unit	Description
Carbon	Influx	Aboveground carbon sequestration	ton ha^−1^ y^−1^	Annual aboveground carbon sequestration rate using census data
Carbon	Influx	Litter carbon production	ton ha^−1^ y^−1^	Product of annual litterfall production and litter carbon concentration using litterfall traps
Carbon	Internal stock	Aboveground carbon stock	ton ha^−1^	Carbon stock in aboveground living and dead biomass using census data
Carbon	Internal stock	Belowground carbon stock	ton ha^−1^	Carbon stock in fine roots and the soil using soil cores
Carbon & Nutrient	Internal flow	Litter decomposition rate	g day^−1^	Daily decomposition rate of leaf litter using litter bags
Carbon	Efflux	Soil respiration	µmol m^−2^ s^−1^	Soil respiration rates using LC-Pro
Water	Internal flow	Water infiltration rate	mm hour^−1^	Water infiltration rate into the water-saturated soil using a metal ring
Water	Internal stock	Maximum soil water content (SWC)	%	5th percentile highest SWC in a year using a TOMST sensor
Water	Internal stock	Minimum SWC	%	95th percentile highest SWC in a year using a TOMST sensor
Water	Internal stock	Intra-annual variation in SWC	%	Coefficient of variation of SWC within a year using a TOMST sensor
Nutrient	Internal flow	Nitrogen (N) resorption rate	%	Difference in N concentration between green and senescent leaves divided by the N concentration of green leaves
Nutrient	Internal flow	Phosphorus (P) resorption rate	%	Difference in P concentration between green and senescent leaves divided by the P concentration of green leaves
Nutrient	Internal flux	Litter N flux	ton ha^−1^ y^−1^	Product of annual litterfall production and litter N concentration
Nutrient	Internal flux	Litter P flux	ton ha^−1^ y^−1^	Product of annual litterfall production and litter P concentration
Nutrient	Internal flow	N mineralization	mg kg^−1^ day^−1^	Mineralization rates of ammonium (NH_4_) and nitrate (NO_3_) using resin balls
Nutrient	Internal flow	P mineralization	mg kg^−1^ day^−1^	Mineralization rates of phosphate (PO_4_) using resin balls

## Material and Methods

### Study Site

In 2021, we established 36 secondary forest plots (25 m × 25 m) on recently abandoned agricultural fields (0–1 year since abandonment), with 17 plots in the tropical dry region and 19 in the tropical wet region in Ghana (Matsuo and others 2023, 2025a). The tropical dry region is located near Abofour in the Ashanti region (7°08′N, 1°45′W). Mean annual precipitation is 1290 mm, with a pronounced dry season (November/December–February), receiving approximately 28 mm per month (Amissah and others 2018). Mean monthly temperatures range from 21.2 to 30.6°C, and soil pH is relatively neutral (pH 5.6–7.8) (Amissah and others 2018; Matsuo and others 2025a). Despite receiving relatively high precipitation compared to some other tropical dry forests, the forests are classified as tropical dry semi-deciduous according to Ghana-wide vegetation classifications (Hall and Swaine 2013).

The tropical wet region is located near Pataho in the Western region (5°09′N, 1°58′W). Mean annual precipitation is higher (1808 mm), with a less severe dry season (82.6 mm per month). Mean monthly temperatures range from 22.8 to 32.0°C, soils are more acidic (pH 4.1–5.6), and forests are classified as tropical wet/moist evergreen (Hall and Swaine 2013). In both regions, secondary forests regenerate rapidly following agricultural abandonment, forming a closed canopy and reaching canopy heights exceeding 10 m within three years.

In 2021, 2022, and 2023, all woody individuals with a stem diameter at breast height (DBH, cm) thicker than 1 cm were identified to species level, and their DBH was measured. We, then, calculated the individual basal area as *π* × (DBH/2)^2^. For multiple-stem individuals, we measured the DBH of only two stems per individual: the largest (DBH_large_, cm) and one average-sized stem (DBH_average_, cm), to reduce the workload instead of measuring all stems (Matsuo and others 2025b). We then counted the total number of stems for each individual (*N*_stems_) and estimated the basal area using the following equation.

1 $$ {\text{Individual basal area}}\, = \,0.{25}\, \times \,\pi \, \times \,\left[ {{\mathrm{DBH}}_{large}^{{2}} \, + \,{\mathrm{DBH}}_{average}^{{2}} \, \times \,\left( {N_{stems} - {1}} \right)} \right] $$

### Soil Laboratory Analysis

In 2021, five soil samples (0–15 cm depth) were taken at the four corners and the center of each plot using an auger, pooled per plot, and analyzed at CSIR-Soil Research Institute of Ghana (SRI) for the following: texture (sand, clay, and silt content, in %); total nitrogen including all forms of organic and inorganic nitrogen (N, in mg g^−1^); plant-available phosphorus (P, in $\mu$ g g^−1^); and total exchangeable bases (TEB, in meq 100 g^−1^) as the sum of exchangeable bases (calcium, magnesium, potassium, and sodium) (Matsuo and others 2024c, 2025a). To obtain soil bulk density (BD, g cm^−3^), additional soil samples (0–15 cm depth) were taken at the same locations using a 5 cm diameter soil ring, oven-dried at 105°C for 120 h, and weighed. BD was calculated as the dry soil mass (excluding pebbles and stones) divided by the volume inside the ring (295 cm^3^).

### Leaf and Stem Traits

To quantify functional composition, we measured functional traits following the standardized protocol (Pérez-Harguindeguy and others 2013; Matsuo and others 2024c). We measured two leaf traits [leaf nitrogen concentration (leaf N, mg g^−1^) and leaf mass per area (LMA, g m^−2^)], and one stem trait [wood density (WD, g cm^−3^)] that are important for carbon, water, and nutrient balance of plants (Reich 2014).

For leaf traits, we measured 65 woody species in dry forests and 104 species in wet forests, which covered, on average, 95.0% of the stand basal area in dry forests (range: 81.9–99.2%) and 98.5% in wet forests (range: 91.7–99.9%). For each species, leaf traits were measured for two sunlit leaves of four or five individuals with a DBH between 1–10 cm and a height up to 8 m, which is a typical size range in early succession.

Wood density data were collected for 77 species in dry forests and 75 species in wet forests, covering, on average, 97.6% of the stand basal area in dry forests (range: 93.7–99.9%) and 97.9% in wet forests (range: 91.2–99.9%). For each species, WD was measured for three individuals with wood cores (4.3 mm diameter) collected with an increment borer (Haglöf Sweden, Langsele, Sweden) or stem slices for small species (DBH < 5 cm) using the water displacement method. WD was calculated as oven-dried mass (at 80°C for 48 h) divided by the fresh volume. This measurement was taken in the study area for 61 species studied; data on WD for the remaining species were taken from the wood density database in Ghana (Djagbletey and others 2020). For detailed methods, see supplementary material (Appendix S1).

### Forest Attributes

We assessed three forest attributes that are important in shaping ecosystem functioning (Poorter and others 2017).

*Forest structure*. We calculated tree density (N, # ha^−1^), indicating tree packing, and stand basal area (BA, m^2^ ha^−1^), indicating the total leaf area (Shinozaki and others 1964).

*Taxonomic diversity*. We calculated taxonomic diversity based on Hill numbers (Chao and others 2014). The first Hill number (^0^D) equals species richness, which is the number of species and is insensitive to relative abundances. The second Hill number (^1^D) equals the exponentiated Shannon–Wiener diversity index and weights species proportionally by their relative abundances. Lastly, we calculated ^1^D/^0^D to quantify species evenness, where values close to 0 indicate a very uneven community and a value of 1 indicates a perfectly even community.

*Functional composition*. We calculated the community-weighted mean (CWM) for each trait (that is representing the trait value of an average-sized species in the community) by multiplying each species’ trait value by its relative basal area within the plot and then summing across all species (Garnier and others 2004). Relative basal area is used instead of abundance because it reflects a species’ biomass, which is an indicator of plant performance and adaptation to local conditions (Lohbeck and others 2013). Species without trait data are excluded from the calculation of CWM.

### Ecosystem Functioning

We measured 16 ecosystem functions related to carbon, water, and nutrient cycling (Table 1). We selected 4–6 functions per cycle to provide a more comprehensive understanding of how ecosystem functions contribute to these cycles and how variable the underlying mechanisms are. For detailed methods, refer to Appendix S2, and for a summary of the values, see Table S1.

*Aboveground carbon sequestration*. Aboveground carbon sequestration was calculated as the difference in aboveground living carbon stock over a one-year interval. Dead trees were subtracted from the aboveground carbon sequestration.

*Litter carbon, nitrogen, and phosphorus fluxes*. Litter was collected using four 0.25 m^2^ litter traps for each plot every month for 7 months (February–August 2023). Litter samples were oven-dried at 65°C for 48 h and weighed for their dry mass (excluding animal feces). A pooled leaf litter sample for each plot was brought to the laboratory at Wageningen University and Research in the Netherlands for the nutrient analysis (C, N, and P). Then, litter carbon, nitrogen, and phosphorus fluxes were calculated as the product of annual litter production and litter nutrient concentration.

*Aboveground carbon stock*. Aboveground carbon stock (AGC stock, ton ha^−1^) was calculated as the sum of carbon stock in the aboveground living and dead biomass. Aboveground living carbon stock (AGC_living_, ton ha^−1^) was calculated by summing the carbon stock of all individuals (≥ 1 cm DBH) following allometric equations developed for Ghanaian secondary tropical forests with a carbon concentration of 0.49 (Becker and others 2012; Addo-Fordjour and Rahmad 2013; Matsuo and others 2025a).

2 $$ {\mathrm{AGC}}_{{{\mathrm{living}}}} \_{\mathrm{tree}}\_{\mathrm{shrub}}\, = \,0.{49}\, \times \,{\text{exp }}\left[ { - {1}.{65}\, + \,{2}.{14}\, \times \,{\mathrm{ln}}\left( {DBH} \right)\, + \,0.{45}\, \times \,{\mathrm{ln}}\left( {WD} \right)} \right] $$

3 $$ {\mathrm{AGC}}_{{{\mathrm{living}}}} \_{\mathrm{liana}}\, = \,0.{49}\, \times \,\left( { - 0.{36}\, + \,{1}.{9}\, \times \,DBH} \right) $$

Aboveground dead carbon stock (AGC_dead_, ton ha^−1^) was estimated by summing the carbon stock of lying deadwood (≥ 5 cm diameter) and standing deadwood (≥ 1 cm diameter) following allometric equations with diameter at the middle point (D_middle_, cm) or DBH, the total length (L, m), a carbon concentration of 0.38, an average decay factor (F = 0.8), and the default shape coefficient (f = 0.5) (Chao and others 2017; Hossain and others 2019; Aghimien and others 2020; Neumann and others 2023).

4 $$ {\mathrm{AGC}}_{{{\mathrm{dead}}}} \_{\mathrm{lying}}\, = \,0.{38}\, \times \,F\, \times \,WD\, \times \,\left( {\pi D_{{{\mathrm{middle}}}}^{{2}} /{4}} \right)\, \times \,L $$

5 $$ {\mathrm{AGC}}_{{{\mathrm{dead}}}} \_{\mathrm{standing}}\, = \,0.{38}\, \times \,F\, \times \,WD\, \times \,f\, \times \,\left( {\pi DBH^{{2}} {/4}} \right)\, \times \,L $$

*Belowground carbon stock*. Belowground carbon stock was estimated as the sum of the carbon stocks in fine roots and the soil. Fine root samples (< 2 mm diameter) were collected based on eight soil cores to a depth of 15 cm per plot and processed following the standard protocol (Freschet and others 2021). To estimate carbon stock in fine roots, fine root biomass was multiplied by the carbon concentration of 0.45 (Huasco and others 2021). Soil organic carbon (SOC) was estimated based on four soil cores (0–15 cm depth) collected at the four corners of each plot in 2023 and multiplied by soil BD to express ton ha^−1^.

*Decomposition rate*. For each forest type, naturally senesced leaf litter collected across all plots was pooled and mixed to prepare litter bags. This approach standardized litter quality among plots within each forest type, thereby allowing decomposition rates to primarily reflect variation in decomposer activity rather than plot-level differences in litter quality. Four litter bags (mesh size of 1.03 mm) containing an initial dry mass of 2 g of leaf litter were incubated for approximately four weeks (April–May 2024) in each plot. Afterward, the remaining litter in the bag was oven-dried at 70°C for 48 h and then weighed. Decomposition rates were calculated as the difference between the weight before and after the incubation, divided by the number of incubation days.

*Soil respiration rate*. Soil respiration rate was measured at three to five locations per plot with a portable soil respiration system (LCpro T, ADC BioScientific Ltd., Hoddesdon, UK) during daytime in January 2024.

*Soil water infiltration rate*. Measurement was taken using a metal tube in March and April 2023. The average of the three infiltration times at soil water saturation was used as the soil water infiltration rate.

*Minimum-, maximum-, and intra-annual variation in soil water content*. Between May 2022 and April 2023, data were recorded every 15 min with a soil moisture logger (TMS-4 datalogger; TOMST s.r.o., Prague, Czech Republic) and calibrated with the HOBO MX Soil Moisture and Temperature Data Logger (Onset Computer, Bourne, MA) to convert the obtained values from TMS4-data logger to volumetric soil water content (m^3^ m^−3^ × 100, that is as %). Then, we calculated the 5th and 95th percentile highest soil water content as the maximum and minimum soil water content, and the coefficient of variation in soil water content within a year as its intra-annual variation.

*Nutrient mineralization rate*. For each plot, nutrient mineralization rates were estimated by installing four resin bags in the soil during the wet season (July–August 2023). Resin bags were prepared by following the protocol (Göransson and others 2016; Jongen and others 2021). After 6 weeks, resin bags were collected and analyzed at CSIR-SRI to determine the mineralization rates of ammonium (NH_4_^+^), nitrate (NO_3_^−^), and phosphate (PO_4_^3−^).

*Nutrient resorption rate*. The nutrient resorption rate was calculated for each plot as CWM green leaf nutrient concentration minus the litter nutrient concentration per plot, divided by the CWM green leaf nutrient concentration multiplied by 100.

### Statistical Analyses

To understand causal and hierarchical relations among environmental conditions, forest attributes, and ecosystem functions, structural equation models (SEM) were used. Our a priori conceptual model (Figure 1) was built based on existing knowledge of this study system (Matsuo and others 2025a). For each driver, we had multiple variables as proxies; six soil variables (bulk density, sand content, clay content, N, P, or TEB), two forest structural variables (tree density or stand basal area), three taxonomic variables (species richness, species evenness, or species diversity), and three functional variables (CWM leaf N, LMA, or WD). Therefore, for each of the 16 ecosystem functions, we ran 108 SEMs testing the different combinations of 6 soil properties × 2 structural variables × 3 taxonomic variables × 3 functional variables to test how different combinations of environmental conditions and forest attributes drive different ecosystem functions. The analysis did not include stand age because of its strong linear relationship with forest structural attributes (Matsuo and others 2025a).

**Figure 2:**
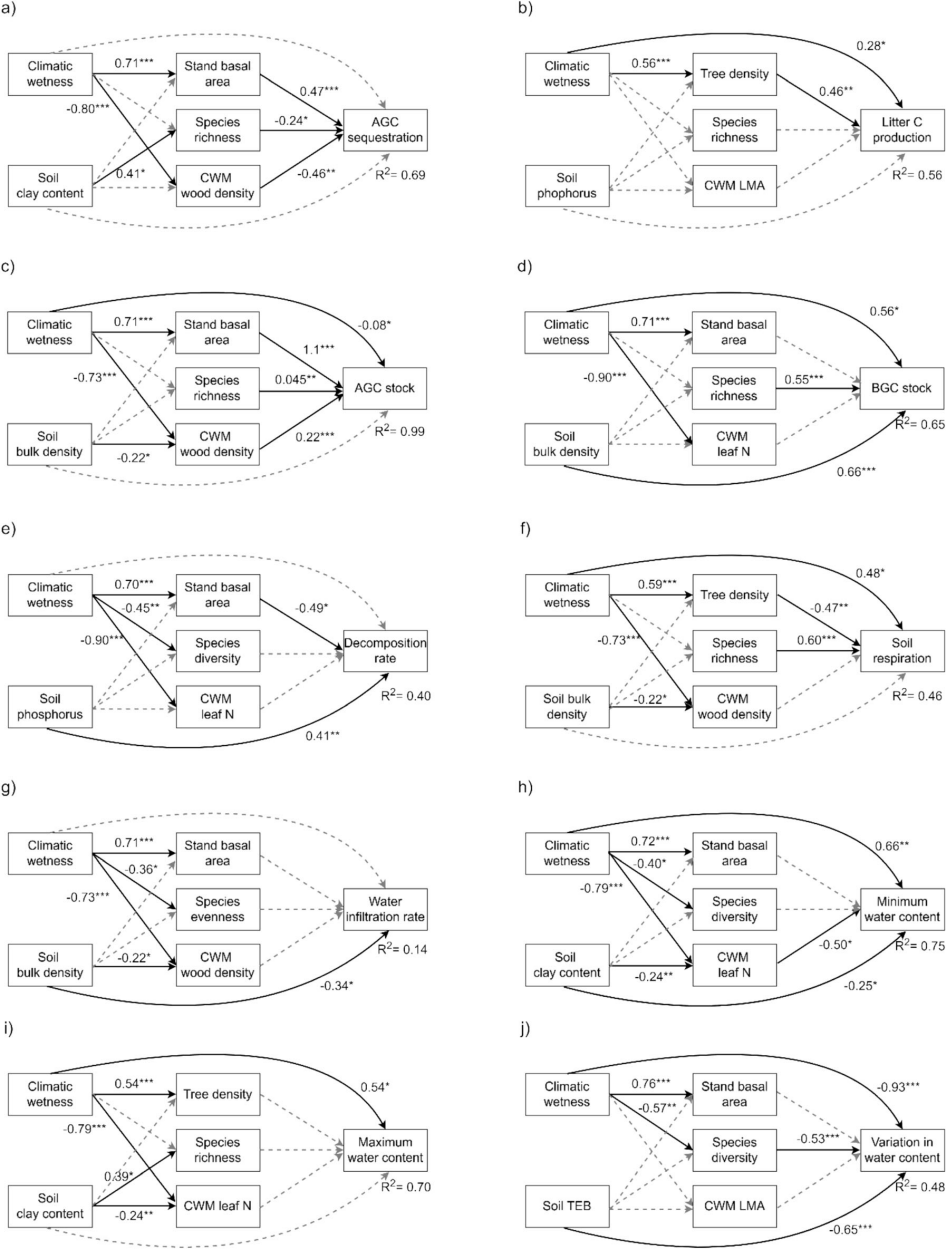

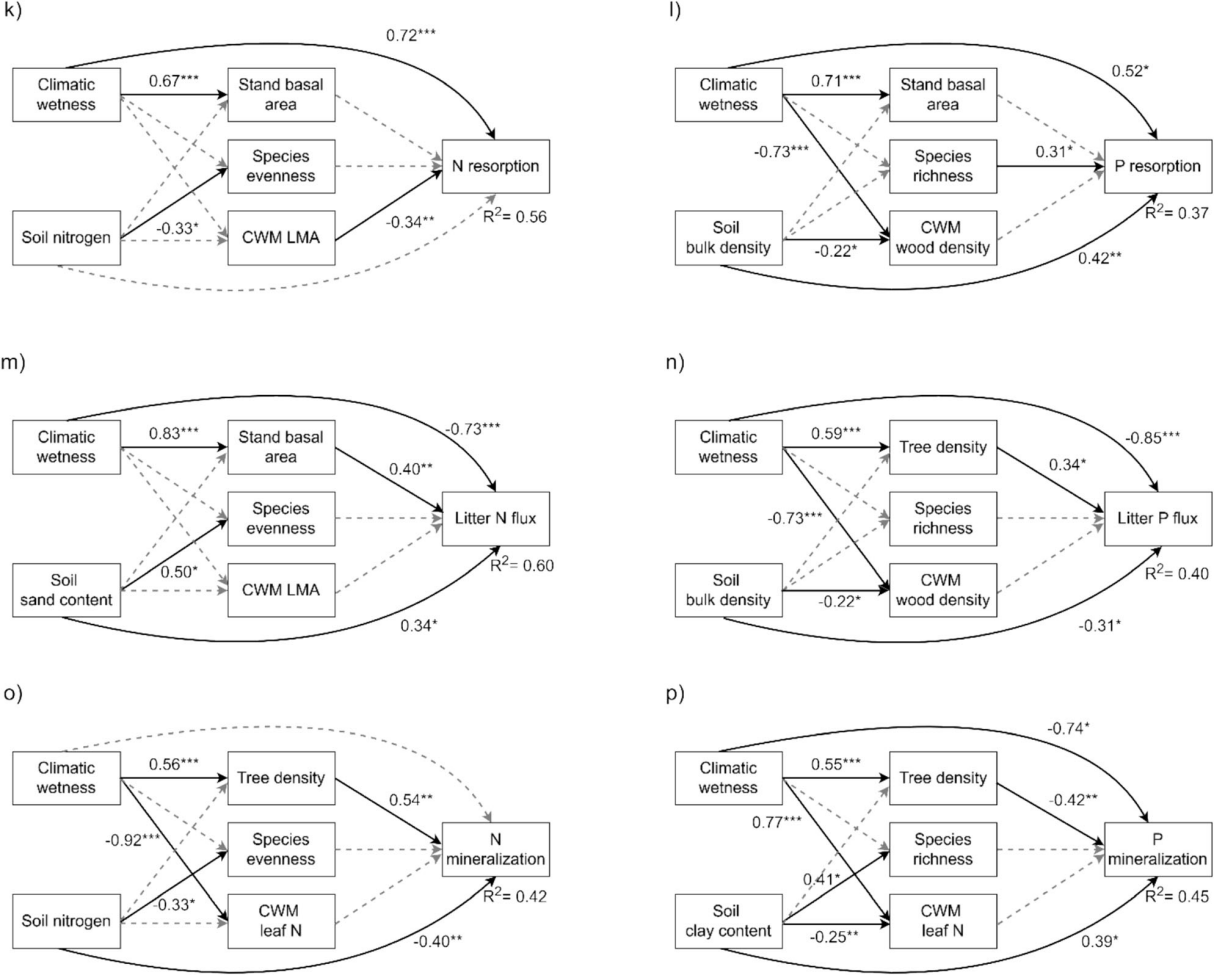
Structural equation models for a) aboveground carbon sequestration (AGC sequestration, ton ha^−1^ y^−1^), b) litter carbon production (Litter C production, ton ha^−1^ y^−1^), c) aboveground carbon stock (AGC stock, ton ha^−1^), d) belowground carbon stock (BGC stock, ton ha^−1^), e) litter decomposition rate (g day^−1^), f) soil respiration rate (µmol m^−2^ s^−1^), g) soil water infiltration rate (mm h^−1^), h) minimum soil water content (%), i) maximum soil water content (%), j) intra-annual variation in soil water content (Variation in water content, %), k) nitrogen resorption rate (N resorption, %), l) phosphorus resorption rate (P resorption, %), m) litter nitrogen flux (Litter N flux, ton ha^−1^ y^−1^), n) litter phosphorus flux (Litter P flux, ton ha^−1^ y^−1^), o) mineralization rate of ammonium and nitrate (N mineralization, mg kg^−1^ day^−1^), and p) mineralization rate of phosphate (P mineralization, mg kg^−1^ day^−1^). Direct and indirect standardized effects of climatic wetness and soil conditions [that is soil bulk density, soil sand or content, soil nitrogen, soil phosphorus, or soil total exchangeable bases (TEB)] and direct effects of forest structure (that is tree density or stand basal area), taxonomic diversity (that is species richness, species evenness, or species diversity), and functional composition [that is a community-weighted mean (CWM) stem or leaf trait] were evaluated. For all relations that were significant (continuous black arrows), the beta coefficient and significance level are given (*p < .05, **p < .01, ***p < .001), and for all non-significant relations (gray, dashed arrows), they are included in the best models but no statistics are shown. R^2^ values show the explained variance of the final response variables (that is ecosystem functioning). For more statistics on the structural equation models, see Table S2.

**Figure 3:**
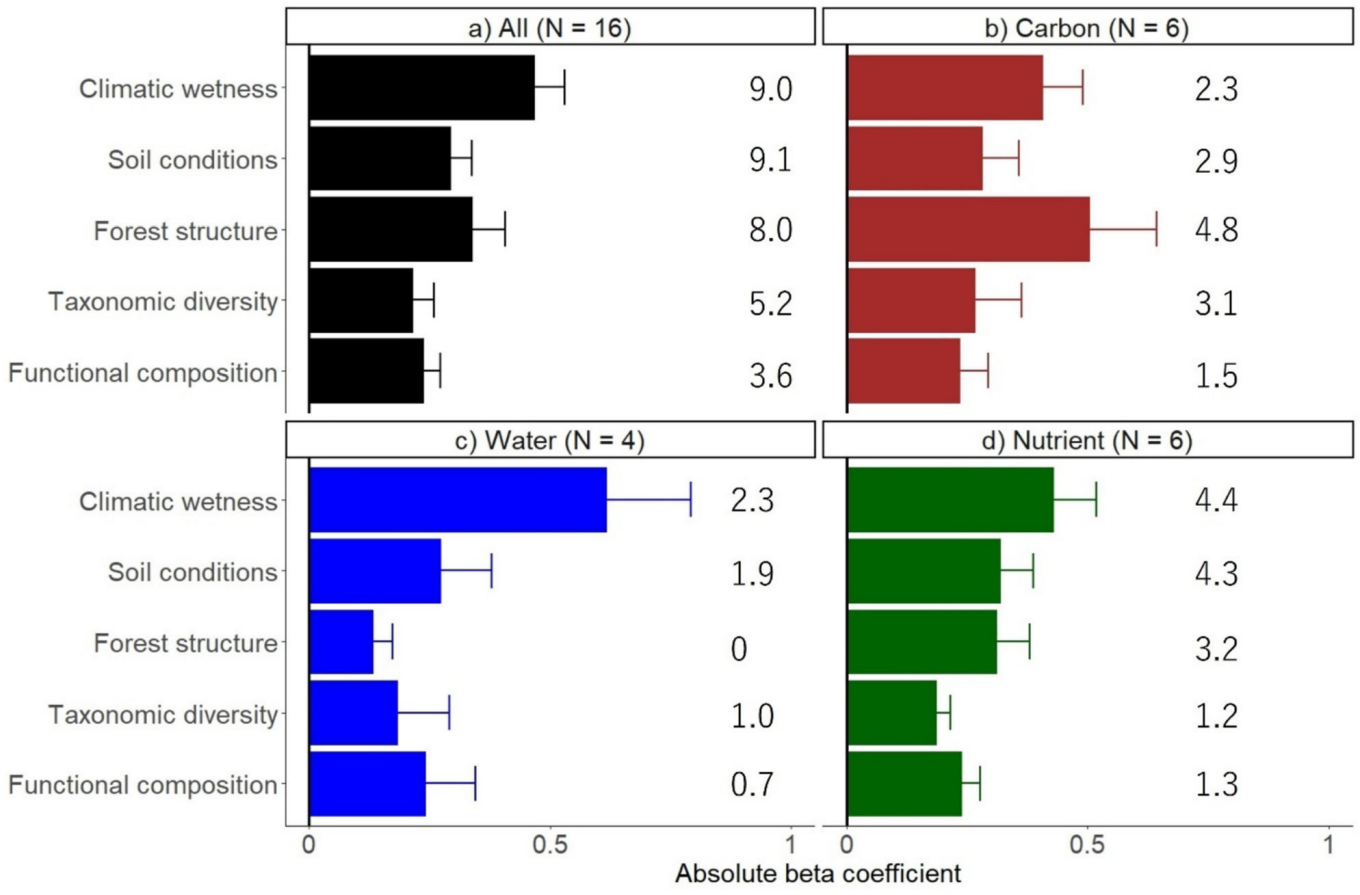
Mean and standard error of the absolute weighted-average standardized effects of each predictor variable—environmental conditions (climatic wetness and soil conditions) and forest attributes (forest structure, taxonomic diversity, and functional composition)—on (a) all 16 ecosystem functions, (b) six carbon functions (aboveground carbon sequestration, litter carbon production, above- or belowground carbon stock, litter decomposition rate, and soil respiration), (c) four water functions (soil water infiltration rate and minimum, maximum, and intra-annual variation in soil water content), and (d) six nutrient functions (nitrogen/phosphorus resorption, litter nitrogen/phosphorus flux, and nitrogen/phosphorus mineralization rates), based on the selected structural equation models (SEM). Numbers next to each bar indicate the number of significant direct effects for each predictor variable based on the selected SEM models (see Methods).

To identify the best combinations of drivers that shape each ecosystem function, we performed the model selection procedure by first rejecting all models with a significantly poor overall fit (*P* < 0.05 from the Chi-square test). Among the remaining models, we selected the best-fitting model based on the model R^2^ for the specific ecosystem function. We used R^2^ of the final response variables (that is ecosystem functions) instead of AIC or BIC for model selection because our primary goal was to identify the best combinations of drivers that shape ecosystem functions rather than to evaluate overall model performance. Additionally, all 108 SEMs that were fitted for each function included the same number of predictors (two environmental variables and three forest attributes), which ensured that model complexity remained constant. This minimizes overfitting and allows R^2^ values to be directly comparable across models. To show the consistency of the results among the top models, we present not only the best model (Figures 2, 3 and Table S2) but also the top three models (Table S3).

To compare the relative importance of five drivers (macroclimate, soil conditions, structure, diversity, and functional composition) in shaping ecosystem functions across the three biogeochemical cycles, we applied a model averaging approach. For each ecosystem function, we identified the best models as those within 95% of the maximum R^2^. For example, if the highest R^2^ for a specific function was 0.75, all models with R^2^ ≥ 0.7125 were included, following the recommendation of using a 95% confidence set of models to account for model selection uncertainty (Grueber and others 2011). We then calculated the weighted averages of the path coefficients for each driver by calculating the path coefficients, with weights proportional to their respective model R^2^ values.

Finally, we calculated the mean and standard error of the absolute path coefficient (that is absolute standardized effects) for each of the five drivers across all 16 ecosystem functions as well as the subsets of six carbon functions, four water functions, and six nutrient functions (Figure 3, Table S4). In this study, rather than calculating a single ecosystem multifunctionality index, we adopted this approach because our primary objectives were (1) to identify the underlying drivers and mechanisms shaping each ecosystem function and (2) to assess the relative importance of different drivers and mechanisms across three biogeochemical cycles. Accordingly, although we calculated the mean of absolute weighted-average standardized effects across all functions, this metric was used to synthesize results rather than to serve as a core ecosystem multifunctionality index. All data analyses were conducted in R (R Core Team 2024), mainly using the following packages *Lavaan* (Rosseel 2012), *ggplot2* (Wickham 2016), and *tidyverse* (Wickham and others 2019).

During the preparation of this work, the authors used ChatGPT (OpenAI) to improve the clarity of the text. After using this tool, the authors carefully reviewed and edited the content, and take full responsibility for the content of the published article.

## Results

We tested our conceptual model (Figure 1) by developing a SEM for each of the 16 ecosystem functions. The selected models explained, on average, 54% of the variation in ecosystem functioning, ranging from 14% for water infiltration rate to 99% for AGC stock (Figure 2). Ecosystem functions were most significantly affected by macroclimate and soil conditions (9 functions each), followed by forest structure (8 functions), taxonomic diversity (5 functions), and functional composition (4 functions) (Figure 3).

**Figure 4:**
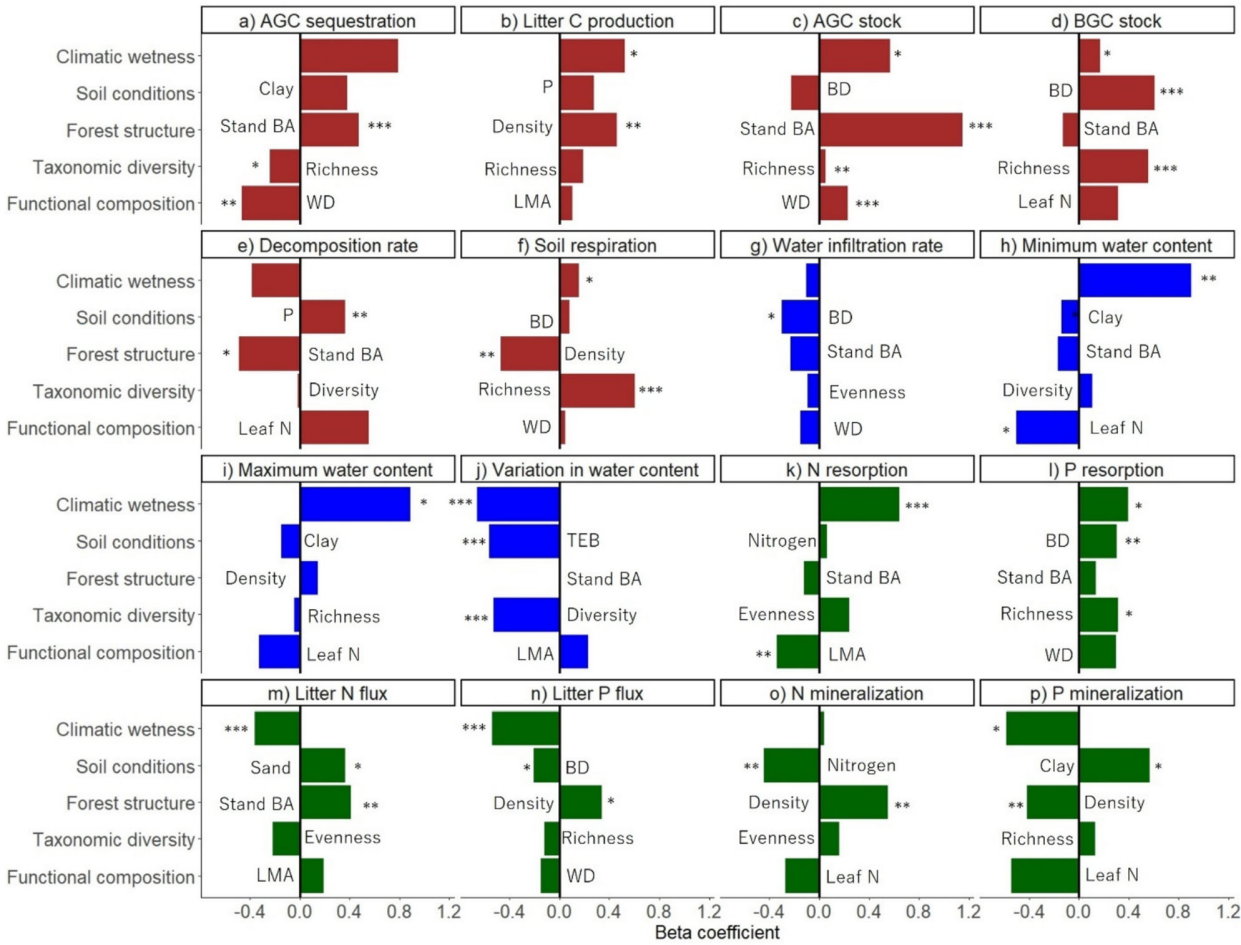
Total standardized effects (sum of direct and indirect standardized effects including the non-significant effects) of each predictor variable (that is environmental conditions: climatic wetness and soil conditions and forest attributes: forest structure, taxonomic diversity, and functional composition) on 16 different ecosystem functions: a) aboveground carbon sequestration (AGC sequestration, ton ha^−1^ y^−1^), b) litter carbon production (Litter C production, ton ha^−1^ y^−1^), c) aboveground carbon stock (AGC stock, ton ha^−1^), d) belowground carbon stock (BGC stock, ton ha^−1^), e) litter decomposition rate (g day^−1^), f) soil respiration rate (µmol m^−2^ s^−1^), g) soil water infiltration rate (mm h^−1^), h) minimum soil water content (%), i) maximum soil water content (%), j) intra-annual variation in soil water content (Variation in water content, %), k) nitrogen resorption rate (N resorption, %), l) phosphorus resorption rate (P resorption, %), m) litter nitrogen flux (Litter N flux, ton ha^−1^ y^−1^), n) litter phosphorus flux (Litter P flux, ton ha^−1^ y^−1^), o) mineralization rate of ammonium and nitrate (N mineralization, mg kg^−1^ day^−1^), and p) mineralization rate of phosphate (P mineralization, mg kg^−1^ day^−1^) based on the selected best structural equation models (Figure 2). Abbreviations are as follows; soil clay content (Clay), soil sand content (Sand), soil bulk density (BD), soil phosphorus (P), soil nitrogen (Nitrogen), soil total exchangeable bases (TEB), stand basal area (Stand BA), tree density (Density), wood density (WD), leaf mass per area (LMA), and leaf nitrogen concentration (Leaf N). The bar graphs are color-coded as follows: carbon functions in brown, water functions in blue, and nutrient functions in dark green. Significant levels of the direct effects based on SEM are given (*P < 0.05; **P < 0.01, ***P < 0.001).

Climatic wetness played a crucial role in shaping multiple ecosystem functions by enhancing above- and belowground carbon sequestration and stocks (Figure 4a, b, c, d), soil respiration (Figure 4f), water content throughout the year (Figure 4h, i, j), and nutrient conservation (Figure 4k, l), while decreasing litter nutrient flux and phosphate mineralization (Figure 4m, n, p). Soil physical properties had more significant direct effects on ecosystem functioning (6 models) than soil nutrients (3 models) (Figure 2). Increased soil compaction (that is high soil BD) increased belowground carbon stock and phosphorus resorption (Figure 4d, l) and reduced water infiltration and litter phosphorus flux (Figure 4g, n). Soil clay content reduced the minimum soil water content (Figure 4h) and increased phosphate mineralization (Figure 4p). In addition, soil sand content increased litter nitrogen flux (Figure 4m). For soil nutrients, soil phosphorus increased litter decomposition rates (Figure 4e), soil TEB reduced intra-annual variation in soil water content (Figure 4j), and soil nitrogen decreased nitrogen mineralization (Figure 4o).

For forest structure, tree density and stand basal area had a similar amount of significant effects on ecosystem functioning. Forest structure increased aboveground carbon sequestration and stock (Figure 4a, b, c) but decreased litter decomposition rates and soil respiration (Figure 4e, f). Although forest structure had consistent positive effects on litter nutrient fluxes (Figure 4m, n), it showed mixed effects on nutrient mineralization (Figure 4o, p). Regarding taxonomic diversity, species richness had the most significant effect on ecosystem functioning (in 5 models) followed by species diversity (exponentiated Shannon–Wiener diversity index, in 1 model), whereas species evenness did not have any significant effect. Species richness increased above- and belowground carbon stocks (Figure 4c, d), soil respiration (Figure 4f), and was associated with higher phosphorus resorption efficiency (Figure 4l), but reduced AGC sequestration rates (Figure 4a), and species diversity reduced intra-annual variation in soil water content (Figure 4j). Functional composition had surprisingly little effect on ecosystem functioning. CWM WD increased AGC stock but reduced AGC sequestration rates (Figure 4a, c). CWM leaf N decreased the minimum soil water content (Figure 4h), and CWM LMA decreased nitrogen resorption rates (Figure 4k). Bivariate scatterplots for all relationships used in the SEMs are shown in Figures S1, S2 and S3.

The relative importance of these drivers and mechanisms varied both within and across biogeochemical cycles (Figure 3, 4). The carbon cycle was most influenced by forest structure and taxonomic diversity (Figure 3b), the water cycle primarily by environmental conditions (particularly climate) (Figure 3c), and the nutrient cycle by a combination of environmental conditions (climate and soils) and forest structure (Figure 3d).

## Discussion

We evaluated how environmental conditions and forest attributes determine 16 ecosystem functions related to three biogeochemical cycles in young secondary tropical forests. We found that 1) environmental conditions and vegetation quantity (forest structure) had stronger effects than vegetation quality (taxonomic diversity and functional composition) in shaping ecosystem multifunctionality, and 2) the relative importance of these drivers varied within and across cycles: The carbon cycle was most influenced by forest structure and taxonomic diversity, whereas the water and nutrient cycles were primarily shaped by environmental conditions (climate and soils), with forest structure additionally shaping nutrient cycling.

### The Relative Importance of Abiotic and Biotic Drivers Varies Across and Within Biogeochemical Cycles

Overall, environmental conditions and forest structure showed the most significant direct effects on ecosystem functioning, supporting the view of the *environmental driver hypothesis* (Wallis and others 2021) and the *vegetation quantity hypothesis* (Lohbeck and others 2015; Teixeira and others 2020). In contrast, taxonomic diversity and functional composition played minor roles (Figure 3). This could be because most pioneer species have relatively similar functional traits due to strong environmental filtering in early succession (van der Sande and others 2024), leading to similar CWM trait values across plots (Tables S5 and S6) and, consequently, weakening niche complementarity. Alternatively, the weak diversity effects we observed in young secondary forests may be due to a relatively low overall plant biomass, making resources less limiting than in mature forests, and hence making species complementarity through resource partitioning less important (Mason and others 2020). However, as succession advances, the relative importance of these drivers may shift, with vegetation quality becoming more important with increased taxonomic and functional diversity (Zheng and others 2024), while vegetation quantity becomes less important as forest structure converges across stands.

Ecosystem functioning across different cycles was driven by different combinations of abiotic and biotic factors. Carbon functions were most strongly shaped by forest attributes (particularly forest structure and taxonomic diversity), which reflect the degree of forest development during secondary succession (van Breugel and others 2006). Water functions were primarily determined by environmental conditions, with climate regulating water input through rainfall and soil properties influencing infiltration and water-holding capacity (Falk and others 2024). Nutrient functions were shaped by both environmental conditions, which affect microbial abundance and activity, and forest structure, which influences litter production and modifies the understory microclimate (Lebrija-Trejos and others 2011; Matsuo and others 2025a, 2025b). Together, these findings underscore the need to consider both abiotic and biotic drivers, as well as multiple ecosystem functions across cycles, to develop a comprehensive understanding of ecosystem multifunctionality in young secondary forests and to guide the restoration of multifunctional tropical forests.

### Climatic Wetness is the Strongest Driver of Ecosystem Functioning

Climatic wetness strongly affected ecosystem functioning because it reflects not only water input but also forest types, growing conditions, and species composition (Figures 3 and S4). For carbon functions, it increased litter carbon production (Figure 2b), possibly through higher branch and leaf production rates because of a longer growing season and intense light competition (Chakravarty and others 2019; Matsuo and others 2024b, 2024a). Increased litterfall production subsequently contributes to soil carbon accumulation (Feng and others 2019; Giweta 2020) and enhances soil respiration by increasing microbial abundance and activities (Yuste and others 2007). Surprisingly, climatic wetness had no additional direct effect on AGC stock and a negative direct effect on AGC sequestration rates (Figure 2c, d). Yet, its strong positive indirect effects through structural development led to overall positive total effects on these functions (Figures 4a, c and S4a, c).

For water functions, climatic wetness increased soil water content throughout the year through larger water input and weaker seasonality (Figure 2h, i, j). For nutrient functions, climatic wetness improved nutrient resorption rate (Figure 2k, l), likely due to the high abundance of evergreen, shade-tolerant, and non-nitrogen-fixing species in wet forests, which generally have high resorption rates (Niinemets and Tamm 2005; Poorter and others 2009; Gei and others 2018). However, high nutrient resorption efficiency reduces litter nutrient concentrations, thereby decreasing litter nutrient fluxes (Figure S5) and constraining microbial activity and mineralization rates by increasing litter C:N and C:P ratios (Schuur and Matson 2001; Schuur and others 2001).

### Soil Physical Properties, Rather than Soil Nutrients, Drive Ecosystem Functioning

Soil physical properties strongly drove various ecosystem functions, particularly belowground functions (Figure 3, 4). Compact soils (that is high soil BD) increased soil carbon stock because of greater soil mass per unit volume, but decreased water infiltration by reducing soil porosity (Figure 2g) (Lulandala and others 2022). This reduced infiltration can lead to increased soil erosion, waterlogging, and nutrient leaching (Falk and others 2024), increasing the need for species to retain the nutrients they have in their tissues and hence promoting N and P resorption (Figure 2l, n) (Lopez and Kursar 2003).

Sandy soils typically have lower water-holding capacity compared to clay soils due to their higher pore density and larger pores. As a result, sandy soils may favor drought-adapted deciduous and nitrogen-fixing species with higher leaf nitrogen concentrations (Matsuo and others 2024c; Xiao and others 2024), facilitating litter nitrogen flux. In contrast, clay soils provide more minerals and support microbes by providing more resources (Dalal 1998), accelerating phosphate mineralization (Figure 2p). Surprisingly, clay soils exhibited lower minimum soil water content, possibly because they can become more compacted and cracked during dry seasons than sandy soils, reducing their ability to recharge and retain water.

Compared to soil physical properties, soil nutrients have relatively minor effects on ecosystem functioning, although they are essential for plants and microbes. Soil phosphorus increased litter decomposition rates, possibly because phosphorus is essential for microbial DNA and ATP, thereby enhancing metabolic activity (Prescott and Vesterdal 2021). Soil TEB reduced intra-annual variation in soil water content (Figure 2j), possibly due to its positive correlations with both clay content and soil organic matter (Helling and others 1964), both of which improve water content. Although previous studies reported positive correlations between soil nutrients and mineralization rates (Li and others 2019; Elrys and others 2021), soil nitrogen concentration decreased nitrogen mineralization. This pattern might be attributed to the high abundance of *Chromolaena odorata* in degraded, low-fertility soils, which increases the abundance of ammonia-oxidizing bacteria in their rhizosphere, thus facilitating nitrogen mineralization (Yuan and others 2024).

### Vegetation Quantity Rather than Quality Drives Ecosystem Functioning

Forest structure was the most important forest attribute in shaping ecosystem functioning (Figures 2, 3). It increased AGC sequestration rates through a larger photosynthetic leaf area (Lehnebach and others 2018), which enhances carbon accumulation over time (Figure S5). The high turnover of branches and leaves leads to greater litter carbon, nitrogen, and phosphorus fluxes (Figure 2b, m, n). However, forest structure reduced belowground functions such as litter decomposition, soil respiration, and phosphate mineralization (Figure 2e, f, p), possibly by reducing the understory irradiance and soil temperature (Lebrija-Trejos and others 2011; Matsuo and others 2021, 2022), which slows down microbial activities (Yuste and others 2007). Forest structure had little impact on water functions (Figure 3), possibly because increased vegetation cover enhances transpiration (Matsuo and others 2025b) while simultaneously reducing soil water evaporation (Lebrija-Trejos and others 2011), or because more developed forests are often dominated by more conservative species with lower transpiration rates (Guillemot and others 2022), resulting in a net neutral effect.

Taxonomic diversity influenced ecosystem functioning in sometimes unexpected ways. While species richness has been shown in other studies to increase productivity through niche complementarity (Cardinale 2011; Brassard and others 2013; van der Plas 2019), we found the opposite pattern in this early successional stage (Figure 2a). This may be because, within our young forests, higher species richness often reflects a comparatively later successional stage with more shade-tolerant species that have inherently slower growth rates (Rüger and others 2023). At the same time, these species have greater survival rates, thus increasing the biomass residence time and AGC stock (Figure 2c). The higher abundance of shade-tolerant species might also contribute to more efficient phosphorus resorption rates (Figure 2l). Moreover, species richness increased belowground carbon stocks, possibly by increasing fine root biomass through spatial niche complementarity in root occupation (Brassard and others 2013) and by providing diverse litter types that contribute to both rapid and long-term soil carbon storage (Freschet and others 2012). The large belowground carbon stocks, as well as these diverse litter types, might also increase microbial abundance and activities, leading to higher soil respiration rates (*energy diversity hypothesis*, Enriquez and others 1993). For water functions, species diversity reduced intra-annual variation in soil water content, probably through spatial and temporal niche complementarity in water use among species.

Functional composition had the fewest significant effects on ecosystem functions, indicating limited support for the *mass-ratio hypothesis* in early succession, possibly due to strong functional similarities among pioneer species (van der Sande and others 2024) and more subtle differences across stands compared to other drivers (Table S6). Community WD reduced AGC sequestration rates (Figure 2a) probably because dense-wooded species have narrower vessels and pit pores, and therefore a lower photosynthetic carbon gain and growth (Santiago and others 2004). However, these species have higher survival rates and longer lifespans (Poorter and others 2010), which positively contribute to AGC stock (Figure 2c). Additionally, these species contain larger biomass for a given stem volume. Community leaf N decreased the minimum soil water content, probably because these species have faster gas exchange rates, increasing transpiration and soil water loss (Guillemot and others 2022). However, the causality may also be reversed, with an increased abundance of nitrogen-fixing species in drier conditions (Gei and others 2018), leading to an observed negative relationship. Community LMA decreased N resorption rates, possibly because nutrients are stored in structurally enforced cell walls, making them more difficult to retract.

## Conclusions

Ecosystem functioning related to carbon, water, and nutrient cycling in young tropical forests is strongly shaped by climatic and edaphic conditions as well as by forest structure, suggesting greater roles for environmental conditions and vegetation quantity over vegetation quality during early succession. This contrasts with findings from mature forests, where vegetation quality often outperforms other drivers (Pelletier and others 2017; Ding and Zang 2021; Noulèkoun and others 2023), indicating a successional shift from a greater relative importance of abiotic drivers and structure in shaping ecosystem multifunctionality in early succession, toward biodiversity later in succession.

Across carbon, water, and nutrient cycles, the relative importance of these drivers varied. The carbon cycle was influenced most strongly by forest attributes, whereas the water and nutrient cycles were primarily shaped by environmental conditions, with forest structure additionally shaping nutrient cycling. Based on these results, we recommend natural regeneration as a scalable, low-cost solution to restore ecosystem multifunctionality, especially in wet tropical forests. Additionally, management interventions can be used to accelerate and steer the recovery of specific biogeochemical cycles. For example, carbon cycling can be enhanced by seeding or planting diverse tree species to accelerate structural development and increase species diversity (Erskine and others 2006); water cycling can be improved by soil scarification to reduce soil bulk density, enhance infiltration, and water-holding capacity (Falk and others 2024); and nutrient cycling can be accelerated through litter additions from neighboring forests, which supply nutrient inputs and stimulate the recovery of the soil microbiome (Wood and others 2009; Sayer and Tanner 2010). Our finding that different drivers influence different cycles also highlights that single management interventions cannot promote all ecosystem functions of interest. Hence, we recommend that future studies assess trade-offs and synergies between ecosystem functions of different cycles, as well as the drivers of overall ecosystem multifunctionality.

In sum, our study advanced understanding of the drivers and mechanisms of ecosystem multifunctionality by analyzing multiple ecosystem functions across biogeochemical cycles in young secondary forests. This mechanistic perspective provides a more comprehensive understanding of ecosystem multifunctionality and offers insight into where and how to restore multifunctional tropical forests through natural regeneration.

## Supplementary Information

Below is the link to the electronic supplementary material.

Supplementary file 1 (DOCX 2001 kb)

## Data Availability

Raw data on 16 ecosystem functions, environmental conditions, and forest attributes are available in DANS (Data Archiving and Networked Services) at 10.17026/LS/RATCZG.

## References

[CR1] Addo-Fordjour P, Rahmad ZB. 2013. Mixed species allometric models for estimating above-ground Liana biomass in tropical primary and secondary forests, Ghana. Int Sch Res Not 2013:1–9.

[CR2] Aghimien EV, Osikabor B, Adedeji MS, Adams OT. 2020. Volume techniques for estimating standing and lying dead wood in Okomu national park, Edo state, Nigeria. Biom Biostat Int J 9:111–116.

[CR3] Amissah L, Mohren GMJ, Kyereh B, Agyeman VK, Poorter L. 2018. Rainfall seasonality and drought performance shape the distribution of tropical tree species in Ghana. Ecol Evol 8:8582–8597. 30250725 10.1002/ece3.4384 PMC6144999

[CR4] Balvanera P, Paz H, Arreola-Villa F, Bhaskar R, Bongers F, Cortés S, del Val E, García-Frapolli E, Gavito ME, González-Esquivel CE, Martínez-Ramos M, Martínez-Yrizar A, Mora F, Naime J, Pascual-Ramírez F, Pérez-Cárdenas N, Ugartechea-Salmerón OA, Siddique I, Suazo-Ortuño I, Swinton SM. 2021. Social ecological dynamics of tropical secondary forests. For Ecol Manage 496.

[CR5] Becker GS, Braun D, Gliniars R, Dalitz H. 2012. Relations between wood variables and how they relate to tree size variables of tropical African tree species. Trees - Str Funct 26:1101–1112.

[CR6] Brassard BW, Chen HYH, Cavard X, Laganière J, Reich PB, Bergeron Y, Paré D, Yuan Z. 2013. Tree species diversity increases fine root productivity through increased soil volume filling. J Ecol 101:210–219.

[CR7] Camenzind T, Hättenschwiler S, Treseder KK, Lehmann A, Rillig MC. 2018. Nutrient limitation of soil microbial processes in tropical forests. Ecol Monogr 88:4–21.

[CR8] Cardinale BJ. 2011. Biodiversity improves water quality through niche partitioning. Nature 472:86–91. 21475199 10.1038/nature09904

[CR9] Chakravarty S, Rai P, Pala NA, Shukla G. 2019. Litter Production and Decomposition in Tropical Forest. In: Bhadouria R, Tripathi S, SrivastavaP P, Singh P, Eds. Handbook of Research on the Conservation and Restoration of Tropical Dry Forests, . IGI Global: Pennsylvania, USA. pp 193–212.

[CR10] Chao A, Gotelli NJ, Hsieh TC, Sander EL, Ma KH, Colwell RK, Ellison AM. 2014. Rarefaction and extrapolation with Hill numbers: A framework for sampling and estimation in species diversity studies. Ecol Monogr 84:45–67.

[CR11] Chao KJ, Chen YS, Song GZM, Chang YM, Sheue CR, Phillips OL, Hsieh CF. 2017. Carbon concentration declines with decay class in tropical forest woody debris. For Ecol Manage 391:75–85.

[CR12] Chazdon RL, Blüthgen N, Brancalion PH, Heinrich V, Bongers F. 2025. Drivers and benefits of natural regeneration in tropical forests. Nat Rev Biodiv :1–17.

[CR13] Dalal RC. 1998. Soil microbial biomass: what do the numbers really mean? Aust J Exp Agric 38:649–665.

[CR14] Ding Y, Zang R. 2021. Determinants of aboveground biomass in forests across three climatic zones in China. For Ecol Manage 482.

[CR15] Djagbletey GD, Adu-Bredu S, DUah-Gyamfi A, Aabeyir R, Djagbletey ED, Akpalu SE, Adeyiga GK, Addo-Danso SD, Brown WH, Dabo J, Amponsah-Manu E. 2020. Wood Density Handbook for some West African Trees.

[CR16] Elrys AS, Ali A, Zhang H, Cheng Y, Zhang J, Cai ZC, Müller C, Chang SX. 2021. Patterns and drivers of global gross nitrogen mineralization in soils. Glob Chang Biol 27:5950–5962. 34407262 10.1111/gcb.15851

[CR17] Enriquez S, Duarte CM, Sand-Jensen K. 1993. Patterns in decomposition rates among photosynthetic organisms: the importance of detritus C :N :P content. Oecologia 94:457–471. 28313985 10.1007/BF00566960

[CR18] Erskine PD, Lamb D, Bristow M. 2006. Tree species diversity and ecosystem function: Can tropical multi-species plantations generate greater productivity? For Ecol Manage 233:205–210.

[CR19] Falk D, Winowiecki LA, Vågen TG, Lohbeck M, Ilstedt U, Muriuki J, Mwaniki A, Tobella AB. 2024. Drivers of field-saturated soil hydraulic conductivity: Implications for restoring degraded tropical landscapes. Science of the Total Environment 907. 10.1016/j.scitotenv.2023.168038 37879476

[CR20] FAO. 2020. Global Forest Resources Assessment.

[CR21] Feng C, Wang Z, Ma Y, Fu S, Chen HYH. 2019. Increased litterfall contributes to carbon and nitrogen accumulation following cessation of anthropogenic disturbances in degraded forests. For Ecol Manage 432:832–839. 10.1016/j.foreco.2018.10.025.

[CR22] Finegan B, Peña-Claros M, de Oliveira A, Ascarrunz N, Bret-Harte MS, Carreño-Rocabado G, Casanoves F, Díaz S, Velepucha PE, Fernandez F, Licona JC, Lorenzo L, Negret BS, Vaz M, Poorter L. 2015. Does functional trait diversity predict above-ground biomass and productivity of tropical forests? Testing three alternative hypotheses. Journal of Ecology 103:191–201.

[CR23] Freschet GT, Aerts R, Cornelissen JHC. 2012. A plant economics spectrum of litter decomposability. Funct Ecol 26:56–65.

[CR24] Freschet GT, Pagès L, Iversen CM, Comas LH, Rewald B, Roumet C, Klimešová J, Zadworny M, Poorter H, Postma JA, Adams TS, Bagniewska-Zadworna A, Bengough AG, Blancaflor EB, Brunner I, Cornelissen JHC, Garnier E, Gessler A, Hobbie SE, Meier IC, Mommer L, Picon-Cochard C, Rose L, Ryser P, Scherer-Lorenzen M, Soudzilovskaia NA, Stokes A, Sun T, Valverde-Barrantes OJ, Weemstra M, Weigelt A, Wurzburger N, York LM, Batterman SA, de Moraes MG, Janeček Š, Lambers H, Salmon V, Tharayil N, McCormack ML. 2021. A starting guide to root ecology: strengthening ecological concepts and standardising root classification, sampling, processing and trait measurements. New Phytologist 232:973–1122. 34608637 10.1111/nph.17572 PMC8518129

[CR25] Garnier E, Cortez J, Billès G, Navas ML, Roumet C, Debussche M, Laurent G, Blanchard A, Aubry D, Bellmann A, Neill C, Toussaint JP. 2004. Plant functional markers capture ecosystem properties during secondary succession. Ecology 85:2630–2637.

[CR26] Gei M, Rozendaal DMA, Poorter L, Bongers F, Sprent JI, Garner MD, Aide TM, Andrade JL, Balvanera P, Becknell JM, Brancalion PHS, Cabral GAL, César RG, Chazdon RL, Cole RJ, Colletta GD, De Jong B, Denslow JS, Dent DH, Dewalt SJ, Dupuy JM, Durán SM, Santo MMDE, Fernandes GW, Nunes YRF, Finegan B, Moser VG, Hall JS, Hernández-Stefanoni JL, Junqueira AB, Kennard D, Lebrija-Trejos E, Letcher SG, Lohbeck M, Marín-Spiotta E, Martínez-Ramos M, Meave JA, Menge DNL, Mora F, Muñoz R, Muscarella R, Ochoa-Gaona S, Orihuela-Belmonte E, Ostertag R, Peña-Claros M, Pérez-García EA, Piotto D, Reich PB, Reyes-García C, Rodríguez-Velázquez J, Romero-Pérez IE, Sanaphre-Villanueva L, Sanchez-Azofeifa A, Schwartz NB, De Almeida AS, Almeida-Cortez JS, Silver W, Moreno VDS, Sullivan BW, Swenson NG, Uriarte M, Van Breugel M, Van Der Wal H, Veloso MDDM, Vester HFM, Vieira ICG, Zimmerman JK, Powers JS. 2018. Legume abundance along successional and rainfall gradients in Neotropical forests. Nat Ecol Evol 2:1104–1111. 29807995 10.1038/s41559-018-0559-6

[CR27] Giweta M. 2020. Role of litter production and its decomposition, and factors affecting the processes in a tropical forest ecosystem: A review. J Ecol Environ 44:1–9.

[CR28] Göransson H, Welc M, Bünemann EK, Christl I, Venterink HO. 2016. Nitrogen and phosphorus availability at early stages of soil development in the Damma glacier forefield, Switzerland; implications for establishment of N2-fixing plants. Plant Soil 404:251–261.

[CR29] Grime JP. 1998. Benefits of plant diversity to ecosystems: immediate, filter and founder effects. Journal of Ecology 86:902–910.

[CR30] Grueber CE, Nakagawa S, Laws RJ, Jamieson IG. 2011. Multimodel inference in ecology and evolution: Challenges and solutions. J Evol Biol 24:699–711. 21272107 10.1111/j.1420-9101.2010.02210.x

[CR31] Guillemot J, Martin-StPaul NK, Bulascoschi L, Poorter L, Morin X, Pinho BX, le Maire G, Bittencourt PRL, Oliveira RS, Bongers F, Brouwer R, Pereira L, Melo GAG, Boonman CCF, Brown KA, Cerabolini BEL, Niinemets Ü, Onoda Y, Schneider JV, Sheremetiev S, Brancalion PHS. 2022. Small and slow is safe: On the drought tolerance of tropical tree species. Glob Chang Biol 28:2622–2638. 35007364 10.1111/gcb.16082

[CR32] Hall JB, Swaine MD. 2013. Distribution and ecology of vascular plants in a tropical rain forest Forest vegetation in Ghana. Springer Science & Business Media.

[CR33] Helling CS, Chesters G, Corey RB. 1964. Contribution of organic matter and clay to soil cation-exchange capacity as affected by the pH of the saturating solution. Soil Sci Soc Am J 28:517–520.

[CR34] Hossain MA, Anik AR, Chakma N, Johnson K, Henry M, Jalal R, Carrillo O, Scott C, Birigazzi L, Akhter M, Iqbal Z. 2019. Estimation Procedures of Indicators and Variables of the Bangladesh Forest Inventory. Dhaka, Bangladesh: Forest Department and Food and Agriculture Organization of the United Nations.

[CR35] Huasco WH, Riutta T, Girardin CAJ, Pacha FH, Vilca BLP, Moore S, Rifai SW, del Aguila-Pasquel J, Murakami AA, Freitag R, Morel AC, Demissie S, Doughty CE, Oliveras I, Cabrera DFG, Baca LD, Amézquita FF, Espejo JES, da Costa ACL, Mendoza EO, Quesada CA, Ondo FE, Ndong JE, Jeffery KJ, Mihindou V, White LJT, Bengone NN, Ibrahim F, Addo-Danso SD, Duah-Gyamfi A, Djagbletey GD, Owusu-Afriyie K, Amissah L, Mbou AT, Marthews TR, Metcalfe DB, Aragão LEO, Marimon-Junior BH, Marimon BS, Majalap N, Adu-Bredu S, Abernethy KA, Silman M, Ewers RM, Meir P, Malhi Y. 2021. Fine root dynamics across pantropical rainforest ecosystems. Glob Chang Biol 27:3657–3680. 33982340 10.1111/gcb.15677

[CR36] Huston M. 1979. A General hypothesis of species diversity. Am Nat 113:81–101.

[CR37] IPBES. 2019. Global assessment report on biodiversity and ecosystem services of the Intergovernmental Science-Policy Platform on Biodiversity and Ecosystem Services. Brondizio ES, Settele J, Díaz S, Ngo HT, Eds. IPBES secretariat, Bonn, Germany

[CR38] Jongen R, Hannula SE, Long JR De, Heinen R, Huberty M, Steinauer K, Bezemer TM. 2021. Plant community legacy effects on nutrient cycling, fungal decomposer communities and decomposition in a temperate grassland. Soil Biol Biochem 163.

[CR39] Lebrija-Trejos E, Pérez-García EA, Meave JA, Bongers F, Poorter L. 2010. Functional traits and environmental filtering drive community assembly in a species-rich tropical system. Ecology 91:386–398. 20392004 10.1890/08-1449.1

[CR40] Lebrija-Trejos E, Pérez-García EA, Meave JA, Poorter L, Bongers F. 2011. Environmental changes during secondary succession in a tropical dry forest in Mexico. J Trop Ecol 27:477–489.

[CR41] Lehnebach R, Beyer R, Letort V, Heuret P. 2018. The pipe model theory half a century on: a review. Ann Bot 121:773–795. 29370362 10.1093/aob/mcx194 PMC5906905

[CR42] Li Z, Tian D, Wang B, Wang J, Wang S, Chen HYH, Xu X, Wang C, He N, Niu S. 2019. Microbes drive global soil nitrogen mineralization and availability. Glob Chang Biol 25:1078–1088. 30589163 10.1111/gcb.14557

[CR43] Lohbeck M, Poorter L, Martínez-ramos M, Bongers F. 2015. Biomass is the main driver of changes in ecosystem process rates during tropical forest succession. Ecology 96:1242–1252. 26236838 10.1890/14-0472.1

[CR44] Lohbeck M, Bongers F, Martinez-Ramos M, Poorter L. 2016. The importance of biodiversity and dominance for multiple ecosystem functions in a human-modifed tropical landscape. Ecology 97:2772–2779. 27859119 10.1002/ecy.1499

[CR45] Lohbeck M, Poorter L, Lebrija-Trejos E, Martinez-Ramos M, Meave JA, Paz H, Pérez-García EA, Romero-Pérez IE, Tauro A, Bongers F. 2013. Successional changes in functional composition contrast for dry and wet tropical forest. Ecology 94:1211–6. http://www.ncbi.nlm.nih.gov/pubmed/23923479 10.1890/12-1850.1 23923479

[CR46] Lopez OR, Kursar TA. 2003. Does flood tolerance explain tree species distribution in tropical seasonally flooded habitats? Oecologia 136:193–204. 12743794 10.1007/s00442-003-1259-7

[CR47] Loreau M. 1998. Separating Sampling and Other Effects in Biodiversity Experiments. Oikos 82:600–602.

[CR48] Lulandala L, Bargués-Tobella A, Masao CA, Nyberg G, Ilstedt U. 2022. Excessive livestock grazing overrides the positive effects of trees on infiltration capacity and modifies preferential flow in dry miombo woodlands. Land Degrad Dev 33:581–595.

[CR49] Mason NWH, Orwin KH, Lambie S, Waugh D, Pronger J, Carmona CP, Mudge P. 2020. Resource-use efficiency drives overyielding via enhanced complementarity. Oecologia 193:995–1010. 32844244 10.1007/s00442-020-04732-7

[CR50] Matsuo T, Martínez-Ramos M, Bongers F, van der Sande MT, Poorter L. 2021. Forest structure drives changes in light heterogeneity during tropical secondary forest succession. J Ecol 109:2871–2884. 34588706 10.1111/1365-2745.13680 PMC8453511

[CR51] Matsuo T, Hiura T, Onoda Y. 2022. Vertical and horizontal light heterogeneity along gradients of secondary succession in cool and warm temperate forests. J Veg Sci 33:e13135. 37274931 10.1111/jvs.13135 PMC10234446

[CR52] Matsuo T, Amissah L, Kok J, Poorter L. 2023. Regeneración natural de bosques tropicales en campos agrícolas abandonados en Ghana. Boletín De La SCME 3:44–53.

[CR53] Matsuo T, Bongers F, Martínez-Ramos M, van der Sande MT, Poorter L. 2024a. Height growth and biomass partitioning during secondary succession differ among forest light strata and successional guilds in a tropical rainforest. Oikos 6:e10486.

[CR54] Matsuo T, Martínez-Ramos M, Onoda Y, Bongers F, Lohbeck M, Poorter L. 2024b. Light competition drives species replacement during secondary tropical forest succession. Oecologia 205:1–11. 38727828 10.1007/s00442-024-05551-w PMC11144147

[CR55] Matsuo T, van der Sande MT, Amissah L, Dabo J, Abdul SM, Poorter L. 2024c. Herbaceous species and dry forest species have more acquisitive leaf traits than woody species and wet forest species. Funct Ecol:194–205.

[CR56] Matsuo T, Poorter L, van der Sande MT, Mohammed Abdul S, Koyiba DW, Opoku J, de Wit B, Kuzee T, Amissah L. 2025a. Drivers of biomass stocks and productivity of tropical secondary forests. Ecology 106:e4488. https://esajournals.onlinelibrary.wiley.com/doi/10.1002/ecy.4488. 10.1002/ecy.4488 PMC11737357 39629674

[CR57] Matsuo T, van der Sande MT, Amissah L, Dabo J, Abdul SM, Poorter L. 2025b. Trait-Based Community Assembly in Early Tropical Forest Succession. J Veg Sci 36.

[CR58] Neumann M, Echeverria S, Hasenauer H. 2023. A simple concept for estimating deadwood carbon in forests. Carbon Manag 14:2197762. 10.1080/17583004.2023.2197762.

[CR59] Niinemets Ü, Tamm Ü. 2005. Species differences in timing of leaf fall and foliage chemistry modify nutrient resorption efficiency in deciduous temperate forest stands. Tree Physiol 25:1001–1014. 15929931 10.1093/treephys/25.8.1001

[CR60] Noulèkoun F, Mensah S, Kim HS, Jo H, Gouwakinnou GN, Houéhanou TD, Mensah M, Naab J, Son Y, Khamzina A. 2023. Tree size diversity is the major driver of aboveground carbon storage in dryland agroforestry parklands. Sci Rep 13. 10.1038/s41598-023-49119-9 PMC10721610 38097646

[CR61] Odum EP. 1969. The Strategy of Ecosystem Development. Science (1979) 164:262–70. 10.1126/science.164.3877.262 5776636

[CR62] Pelletier J, Siampale A, Legendre P, Jantz P, Laporte NT, Goetz SJ. 2017. Human and natural controls of the variation in aboveground tree biomass in African dry tropical forests. Ecol Appl 27:1578–1593. 28374945 10.1002/eap.1550

[CR63] Pérez-Harguindeguy N, Díaz S, Garnier E, Lavorel S, Poorter H, Jaureguiberry P, Bret-Harte MS, Cornwell WK, Craine JM, Gurvich DE, Urcelay C, Veneklaas EJ, Reich PB, Poorter L, Wright IJ, Ray P, Enrico L, Pausas JG, De Vos AC, Buchmann N, Funes G, Quétier F, Hodgson JG, Thompson K, Morgan HD, Ter Steege H, Van Der Heijden MGA, Sack L, Blonder B, Poschlod P, Vaieretti MV, Conti G, Staver AC, Aquino S, Cornelissen JHC. 2013. New handbook for standardised measurement of plant functional traits worldwide. Aust J Bot 61:167–234.

[CR64] Poorter H, Niinemets Ü, Poorter L, Wright IJ, Villar R, Niinemets U, Poorter L, Wright IJ, Villar R. 2009. Causes and consequences of variation in leaf mass per area (LMA):a meta-analysis. New Phytol 182:565–588. 19434804 10.1111/j.1469-8137.2009.02830.x

[CR65] Poorter L, McDonald I, Alarcón A, Fichtler E, Licona JC, Peña-Claros M, Sterck F, Villegas Z, Sass-Klaassen U. 2010. The importance of wood traits and hydraulic conductance for the performance and life history strategies of 42 rainforest tree species. New Phytol 185:481–492. 19925555 10.1111/j.1469-8137.2009.03092.x

[CR66] Poorter L, van der Sande MT, Arets EJMM, Ascarrunz N, Enquist B, Finegan B, Licona JC, Martínez-Ramos M, Mazzei L, Meave JA, Muñoz R, Nytch CJ, de Oliveira AA, Pérez-García EA, Prado-Junior J, Rodríguez-Velázques J, Ruschel AR, Salgado-Negret B, Schiavini I, Swenson NG, Tenorio EA, Thompson J, Toledo M, Uriarte M, van der Hout P, Zimmerman JK, Peña-Claros M. 2017. Biodiversity and climate determine the functioning of Neotropical forests. Global Ecol Biogeogr 26:1423–1434.

[CR67] Poorter L, van der Sande MT, Amissah L, Bongers F, Hordijk I, Kok J, Laurance SGW, Martínez-Ramos M, Matsuo T, Meave JA, Muñoz R, Peña-Claros M, van Breugel M, Herault B, Jakovac CC, Lebrija-Trejos E, Norden N, Lohbeck M. 2024. A comprehensive framework for vegetation succession. Ecosphere 15:e4794.

[CR68] Poorter L, van der Sande MT, Thompson J, Arets EJMM, Alarcón A, Álvarez-Sánchez J, Ascarrunz N, Balvanera P, Barajas-Guzmán G, Boit A, Bongers F, Carvalho FA, Casanoves F, Cornejo-Tenorio G, Costa FRC, de Castilho C V., Duivenvoorden JF, Dutrieux LP, Enquist BJ, Fernández-Méndez F, Finegan B, Gormley LHL, Healey JR, Hoosbeek MR, Ibarra-Manríquez G, Junqueira AB, Levis C, Licona JC, Lisboa LS, Magnusson WE, Martínez-Ramos M, Martínez-Yrizar A, Martorano LG, Maskell LC, Mazzei L, Meave JA, Mora F, Muñoz R, Nytch C, Pansonato MP, Parr TW, Paz H, Pérez-García EA, Rentería LY, Rodríguez-Velazquez J, Rozendaal DMA, Ruschel AR, Sakschewski B, Salgado-Negret B, Schietti J, Simões M, Sinclair FL, Souza PF, Souza FC, Stropp J, ter Steege H, Swenson NG, Thonicke K, Toledo M, Uriarte M, van der Hout P, Walker P, Zamora N, Peña-Claros M. 2015. Diversity enhances carbon storage in tropical forests. Glob Ecol Biogeogr 24.

[CR69] Prescott CE, Vesterdal L. 2021. Decomposition and transformations along the continuum from litter to soil organic matter in forest soils. For Ecol Manage 498:119522. 10.1016/j.foreco.2021.119522.

[CR70] R Core Team. 2024. R: a language and environment for statistical computing.

[CR71] Ratcliffe S, Wirth C, Jucker T, van der Plas F, Scherer-Lorenzen M, Verheyen K, Allan E, Benavides R, Bruelheide H, Ohse B, Paquette A, Ampoorter E, Bastias CC, Bauhus J, Bonal D, Bouriaud O, Bussotti F, Carnol M, Castagneyrol B, Chećko E, Dawud SM, De Wandeler H, Domisch T, Finér L, Fischer M, Fotelli M, Gessler A, Granier A, Grossiord C, Guyot V, Haase J, Hättenschwiler S, Jactel H, Jaroszewicz B, Joly FX, Kambach S, Kolb S, Koricheva J, Liebersgesell M, Milligan H, Müller S, Muys B, Nguyen D, Nock C, Pollastrini M, Purschke O, Radoglou K, Raulund-Rasmussen K, Roger F, Ruiz-Benito P, Seidl R, Selvi F, Seiferling I, Stenlid J, Valladares F, Vesterdal L, Baeten L. 2017. Biodiversity and ecosystem functioning relations in European forests depend on environmental context. Ecol Lett 20:1414–1426. 28925074 10.1111/ele.12849

[CR72] Reich PB. 2014. The world-wide ‘fast-slow’ plant economics spectrum: a traits manifesto. Journal of Ecology 102:275–301.

[CR73] Rosseel Y. 2012. lavaan: an R package for structural equation modeling. J Stat Softw 48:1–36.

[CR74] Rüger N, Schorn ME, Kambach S, Chazdon RL, Farrior CE, Meave JA, Muñoz R, Hérault B, Jakovac CC, Norden N, Poorter L, Van Der Sande MT, Wirth C, Delgado D, Dent DH, Dewalt SJ, Dupuy JM, Finegan B, Hernández- JSHJL, Lopez SOR. 2023. Successional shifts in tree demographic strategies in wet and dry Neotropical forests. Glob Ecol Biogeogr 32:1002–1014.

[CR75] van der Sande MT, Poorter L, Derroire G, do Espirito Santo MM, Lohbeck M, Müller SC, Bhaskar R, van Breugel M, Dupuy-Rada JM, Durán SM, Jakovac CC, Paz H, Rozendaal DMA, Brancalion P, Craven D, Mora Ardilla F, Almeida JS, Balvanera P, Becknell J, Finegan B, César RG, Hernández-Stefanoni JL, Kennard D, Letcher SG, Marín-Spiotta E, Muñoz R, Reyes-García C, Sanaphre-Villanueva L, Utrera LP, Fernandes GW, Álvarez FS, Andrade JL, Arreola F, Boukili V, Cabral GAL, Chave J, Chazdon R, Colletta G, das Dores Magalhães Veloso M, de Jong B, Lebrija-Trejos E, de Souza Moreno V, Dent DH, DeWalt S, García ED, Ferreira Nunes YR, Granda V, Hall J, Lobo R, Lopez O, Martínez Ramos M, Meave JA, Ochoa-Gaona S, Sampaio EVSB, Sanchez-Azofeifa A, Teixeira HM, Toledo M, Uriarte M, Wright SJ, Zanini K, Bongers F. 2024. Tropical forest succession increases tree taxonomic and functional tree richness but decreases evenness. Glob Ecol Biogeogr.

[CR76] Santiago LS, Goldstein G, Meinzer FC, Fisher JB, Machado K, Woodruff D, Jones T. 2004. Leaf photosynthetic traits scale with hydraulic conductivity and wood density in Panamanian forest canopy trees. Oecologia 140:543–550. 15232729 10.1007/s00442-004-1624-1

[CR77] Sayer EJ, Tanner EVJ. 2010. Experimental investigation of the importance of litterfall in lowland semi-evergreen tropical forest nutrient cycling. J Ecol 98:1052–1062.

[CR78] Schnitzer SA, Klironomos JN, HilleRisLambers J, Kinkel LL, Reich PB, Xiao K, Rillig MC, Sikes BA, Callway RM, Mangan SA, van Nes EH, Scheffer M. 2011. Soil microbes drive the classic plant diversity–productivity pattern. Ecology 92:296–303. 21618909 10.1890/10-0773.1

[CR79] Schuur EA, Matson PA. 2001. Net primary productivity and nutrient cycling across a mesic to wet precipitation gradient in Hawaiian montane forest. Oecologia 128:431–442. 24549913 10.1007/s004420100671

[CR80] Schuur EAG, Chadwick OA, Matson PA. 2001. Carbon cycling and soil carbon storage in mesic to wet Hawaiian montane forests. Ecology 82:3182–3196.

[CR81] Shinozaki K, Yoda K, Hozumi K, Kira T. 1964. A quantitative analysis of plant form - the pipe model theory I. Basic analysis. Jpn J Ecol 14:97–105.

[CR82] Teixeira HM, Cardoso IM, Bianchi FJJA, da Cruz Silva A, Jamme D, Peña-Claros M. 2020. Linking vegetation and soil functions during secondary forest succession in the Atlantic forest. For Ecol Manage 457:117696. 10.1016/j.foreco.2019.117696.

[CR83] Tilman D. 1999. The ecological consequences of changes in biodiversity: a search for general principles. Ecology 80:1455–1474.

[CR84] van Breugel M, Martínez-Ramos M, Bongers F. 2006. Community dynamics during early secondary succession in Mexican tropical rain forests. J Trop Ecol 22:663–674.

[CR85] van der Plas F. 2019. Biodiversity and ecosystem functioning in naturally assembled communities. Biol Rev 94:1220–1245. 30724447 10.1111/brv.12499

[CR86] van der Sande MT, Peña-Claros M, Ascarrunz N, Arets EJMM, Licona JC, Toledo M, Poorter L. 2017. Abiotic and biotic drivers of biomass change in a Neotropical forest. J Ecol 105:1223–1234.

[CR87] Wallis CIB, Tiede YC, Beck E, Böhning-Gaese K, Brandl R, Donoso DA, Espinosa CI, Fries A, Homeier J, Inclan D, Leuschner C, Maraun M, Mikolajewski K, Neuschulz EL, Scheu S, Schleuning M, Suárez JP, Tinoco BA, Farwig N, Bendix J. 2021. Biodiversity and ecosystem functions depend on environmental conditions and resources rather than the geodiversity of a tropical biodiversity hotspot. Sci Rep 11. 10.1038/s41598-021-03488-1 PMC8720099 34972835

[CR88] Wickham H. 2016. ggplot2 Elegant Graphics for Data Analysis. New York: Springer.

[CR89] Wickham H, Averick M, Bryan J, Chang W, McGowan L, François R, Grolemund G, Hayes A, Henry L, Hester J, Kuhn M, Pedersen T, Miller E, Bache S, Müller K, Ooms J, Robinson D, Seidel D, Spinu V, Takahashi K, Vaughan D, Wilke C, Woo K, Yutani H. 2019. Welcome to the Tidyverse. J Open Sour Softw 4:1686.

[CR90] Wood TE, Lawrence D, Clark DA, Chazdon RL. 2009. Rain forest nutrient cycling and productivity in response to large-scale litter manipulation. Ecology 90:109–121. 19294918 10.1890/07-1146.1

[CR91] Xiao Y, Yang D, Zhang SB, Mo YX, Dong YY, Wang KF, He LY, Dong B, Dossa GGO, Zhang JL. 2024. Nitrogen-fixing and non-nitrogen-fixing legume plants differ in leaf nutrient concentrations and relationships between photosynthetic and hydraulic traits. Tree Physiol 44(5):tpae048. 38691446 10.1093/treephys/tpae048

[CR92] Yuan C, Gao J, Huang L, Jian S. 2024. Chromolaena odorata affects soil nitrogen transformations and competition in tropical coral islands by altering soil ammonia oxidizing microbes. Sci Tot Environ 950:175196. 10.1016/j.scitotenv.2024.175196 39097027

[CR93] Yuste JC, Baldocchi DD, Gershenson A, Goldstein A, Misson L, Wong S. 2007. Microbial soil respiration and its dependency on carbon inputs, soil temperature and moisture. Glob Chang Biol 13:2018–2035.

[CR94] Zheng L, Barry KE, Guerrero-Ramírez NR, Craven D, Reich PB, Verheyen K, Scherer-Lorenzen M, Eisenhauer N, Barsoum N, Bauhus J, Bruelheide H, Cavender-Bares J, Dolezal J, Auge H, Fagundes MV, Ferlian O, Fiedler S, Forrester DI, Ganade G, Gebauer T, Haase J, Hajek P, Hector A, Hérault B, Hölscher D, Hulvey KB, Irawan B, Jactel H, Koricheva J, Kreft H, Lanta V, Leps J, Mereu S, Messier C, Montagnini F, Mörsdorf M, Müller S, Muys B, Nock CA, Paquette A, Parker WC, Parker JD, Parrotta JA, Paterno GB, Perring MP, Piotto D, Wayne Polley H, Ponette Q, Potvin C, Quosh J, Rewald B, Godbold DL, van Ruijven J, Standish RJ, Stefanski A, Sundawati L, Urgoiti J, Williams LJ, Wilsey BJ, Yang B, Zhang L, Zhao Z, Yang Y, Sandén H, Ebeling A, Schmid B, Fischer M, Kotowska MM, Palmborg C, Tilman D, Yan E, Hautier Y. 2024. Effects of plant diversity on productivity strengthen over time due to trait-dependent shifts in species overyielding. Nat Commun 15(1):2078. 38453933 10.1038/s41467-024-46355-z PMC10920907

