# Supplementary material for: Drivers and Mechanisms of Ecosystem Multifunctionality in Secondary Tropical Forests
Source: Ecosystems. 2026 Feb 17;29(2):28. doi: 10.1007/s10021-026-01047-1 (PMC12913265; doi:10.1007/s10021-026-01047-1)
Supplement: Supplementary file 1 — (DOCX 2001 kb) [file 10021_2026_1047_MOESM1_ESM.docx]

**Drivers and mechanisms of ecosystem multifunctionality in secondary tropical forests**

Tomonari Matsuo^1*^, Lucy Amissah^2,3^, Masha T. van der Sande^1^, Fons van der Plas^4^, Jazz Kok^1^, Salim Mohammed Abdul^2^, Lucas Chojnacki^1^, Tijs Kuzee^1^, Lhouyangdar Khulpu^1^, Lourens Poorter^1^

^1^Forest Ecology and Forest Management Group, Wageningen University, Wageningen, the Netherlands.

^2^CSIR-Forestry Research Institute of Ghana, Kumasi, Ghana

^3^CSIR College of Science and Technology, P.O. Box M 32, Accra

^4^Plant Ecology and Nature Conservation Group, Wageningen University, Wageningen, the Netherlands.

* Corresponding author: Tomonari Matsuo ([tomonari.matsuo@wur.nl](mailto:Tomonari.matsuo@wur.nl))

**Appendix S1: Detailed methods on leaf and stem trait measurements.**

For each species, leaf traits were measured on two sunlit leaves from four to five young woody plants. The DBH of sampled plants ranged from 1 to 10 cm, with their height up to 8 m, which is typical size in early successional forests. Leaf samples were collected from outside of the permanent plots using machetes, tall pole pruners, and slingshots. The leaf collection was done during the wet season (between June and July in 2021 and April in 2022). Leaves were scanned to estimate leaf area (LA, cm^2^) using pixel counting software ImageJ (National Institutes of Health, Bethesda, MD, USA), dried in the oven to constant weight (at 70 °C for 48 hours), and weighed to determine leaf dry mass (g). Leaf mass per area (LMA, g cm^-2^) was then calculated as leaf dry mass divided by leaf area. Leaf nitrogen concentration (LNC, mg g^-1^) was measured in the laboratory at Wageningen University & Research in the Netherlands. Oven-dried leaves were digested using a mixture of H_2_SO_4_–Se and salicylic acid (Novozamsky et al., 1983). The digestion process begins with H_2_O_2_, which oxidizes most of the organic matter. After decomposing the excess H_2_O_2_ and evaporating water, the digestion is completed with concentrated H_2_SO_4_ at an elevated temperature (330°C) using Se as a catalyst. Total N in the leaf digests was measured spectrophotometrically with a segmented-flow system (Skalar San++ System). The petiole was excluded from leaf trait measurements.

Wood density was based on wood cores (0.43 cm diameter), using an increment borer (Haglöf Sweden, Langsele, Sweden), and fresh volume was calculated with its diameter (0.43 cm) and length (L, cm) (eq. 1)

Fresh volume = π × (*d/*4*)^2^* × *L* (eq. 1)

Stem slices were sampled for species with small stems (DBH<5 cm). For those samples, the fresh volume was determined with the water displacement method. Wood density (WD, g cm^-3^) was calculated as oven-dried mass (at 80 ℃ for 48 hours) over fresh volume. This measurement was taken in the study area for 61 species studied; data on WD for the remaining species were taken from the wood density database in Ghana (Djagbletey et al., 2020). For each species, stem samples were collected from three adult individuals at the beginning of the wet season (March 2023). All traits were measured following standardized protocols (Pérez-Harguindeguy et al., 2013).

**Appendix S2: Detailed methods on the measurements of ecosystem functioning**

*Aboveground carbon productivity*

Aboveground carbon productivity (ton ha^-1^ year^-1^) was calculated as the difference in aboveground carbon stock over one year.

*Litter carbon, nitrogen, and phosphorus fluxes*

The plots were subdivided into four quadrants (12.5 m × 12.5 m), and one litter trap (50 × 50 cm, at a height of 1.0 m) was placed at the center of each quadrant (i.e., four traps per plot). To estimate the annual litter production (ton ha^-1^ year^-1^), litter was collected every month for 7 months (February-August in 2023), which covers two months of dry season and five months of wet season, which is considered as a sufficient sampling effort to estimate to annual litter production. Each month, litter samples were collected and separated into leaf materials (leaves and petioles), branches, reproductive parts (flowers, fruits, and seeds), and animal faceas (from lizards, bats, and birds that defecated in the traps). Afterward, these components were oven-dried at 65 ℃ for 48 hours and weighed for their dry mass. Because we were only interested in the forest litter production rate, we only used the sum of plant materials for the litter production rate. The weight of litter was summed for each plot and multiplied by 10,000 to express it per ha.

A pooled leaf litter sample for each plot was then brought to the laboratory at Wageningen University & Research in the Netherlands for nutrient analysis (C, N, and P). Carbon content was analyzed by using a CHN analyzer, where the sample was combusted in an oxygen-rich environment, and the resulting CO₂ was quantified to determine the total carbon content. For N and P, oven-dried leaves were digested using a mixture of H_2_SO_4_–Se and salicylic acid (Novozamsky et al., 1983). The digestion process begins with H_2_O_2_, which oxidizes most of the organic matter. After decomposing the excess H_2_O_2_ and evaporating water, the digestion is completed with concentrated H_2_SO_4_ at an elevated temperature (330°C) using Se as a catalyst. Total N and P in the leaf digests were then measured spectrophotometrically with a segmented flow system (Skalar San++ System). Then, litter carbon, nitrogen, and phosphorus fluxes (ton ha^-1^ year^-1^) were calculated as the product of annual litter production and litter nutrient concentration.

*Aboveground carbon stock*

Aboveground carbon stock (AGC stock, ton ha^-1^) was estimated as the sum of carbon stock in the aboveground living and dead biomass. Aboveground living carbon stock (AGC_living_, ton ha^-1^) was calculated by summing the carbon stock of all individuals (≥1 cm DBH) following allometric equations developed for Ghanaian tropical secondary forests with a carbon concentration of 0.49 (eq. 2,3) (Addo-Fordjour & Rahmad, 2013; Becker et al., 2012; Matsuo, 2024). When the wood density (WD, g cm^-3^) data for some species was not available, we used local wood density data at the highest taxonomic resolution available (genus-level or family-level) or the average wood density for each site.

AGC_living__tree_shrub = 0.49 × exp [-1.65 + 2.14 × ln(*DBH*) + 0.45 × ln(*WD*)] (eq.1)

AGC_living__liana = 0.49 × (-0.36 + 1.9 × *DBH*) (eq. 2)

Aboveground dead carbon stock (AGC_dead_, ton ha^-1^) was estimated by summing the carbon stock of lying deadwood (≥5 cm diameter) and standing deadwood (≥1 cm diameter). Dead wood lying on the ground was inventoried along two parallel 25 m long transects within each plot that were spaced 15 m apart, using the line-intercept method. The diameter of all deadwoods bisecting a transect was recorded, along with its diameter at mid-point (D_middle_, cm) and length (L, m). All standing deadwood with DBH > 1cm was inventoried throughout each plot, with DBH and L recorded. To estimate their biomass, we used the following equations for lying deadwood (eq. 3, Aghimien et al., 2020) or for standing deadwood (eq. 4) with the default shape coefficient (f=0.5, Puletti et al., 2019), the average decay factor (F=0.8, Hossain et al., 2019), and a carbon concentration of 0.38 (Chao et al., 2017).

AGC_dead__lying = 0.38 × *F* × *WD* × (π*D* _middle_^2^ / 4) × *L* (eq.3)

AGC_dead__standing = 0.38 × *F* × *WD* × *f* × (π*DBH*^2^ / 4) × *L* (eq. 4)

Carbon stock in aboveground lying dead biomass per plot was calculated as the sum of all lying deadwood carbon stock within the 50 m^2^ (= 25 m^2^ × 2) transect and then multiplied by 200 to express it per ha. Similarly, carbon stock in aboveground standing dead biomass per plot was calculated as the sum of all standing deadwood carbon stock and multiplied by 16 to express it per ha.

*Belowground carbon stock*

Belowground carbon stock was estimated as the sum of carbon stocks in fine roots and soil. In April and May 2023, soil cores were taken to a depth of 15 cm using a 5 cm diameter soil ring to estimate carbon stocks in the fine roots and soil. Because in many places rocks were present beyond 15 cm soil depth, sampling was limited to this depth. Additionally, this is the depth at which soil organic carbon (SOC) is most strongly affected by litter production (Feng et al., 2019) and thus changes most rapidly during succession (van der Sande et al., 2022). To account for spatial heterogeneity, for each plot, eight samples were taken for fine root biomass and four samples for SOC. SOC was determined by the modified dichromate oxidation method of Walkley-Black (Nelson & Sommers, 1983), and then multiplied by soil BD to express ton ha^-1^. Root samples were processed following the standard protocol (Freschet et al., 2021). Root samples were properly washed after soaking them in water for up to 24 hours, then sieved with a 0.25 mm mesh sieve, and oven-dried at 65°C for 48 hours. Samples were then separated into fine (<2mm diameter) and coarse (>2mm) roots, which were weighed separately. We only used the data of fine roots for the analysis. To estimate carbon stock in fine roots, fine root biomass was multiplied by the carbon concentration of 0.45 (Huaraca Huasco et al., 2021).

*Decomposition rate*

Litter decomposition rates were estimated using the litterbag method. For each forest type, naturally senesced leaf litter collected across all plots was pooled and mixed to prepare forest-type–specific litter bags. This approach standardized litter quality among plots within each forest type, thereby allowing decomposition rates to primarily reflect variation in decomposer activity rather than plot-level differences in litter quality. Four litter bags (mesh size of 1.03 mm) containing an initial dry mass of 2 grams of mixed litter were incubated for approximately four weeks (April-May 2024) in each plot. Afterward, the remaining litter in the bag was carefully brushed, oven-dried at 70 ℃ for 48 hours, and then weighed. Decomposition rates were assumed to be linear because of the relatively short incubation period, and calculated as the difference between the weight before and after the incubation divided by the number of incubation days.

*Soil respiration rate*

Soil respiration rate was measured with a portable soil respiration system (LCpro T, ADC BioScientific Ltd., Hoddesdon, UK) fitted with a soil respiration chamber (LCPro Soil Hood, ADC BioScientific Ltd., UK) during the daytime (from 06:30 to 17:30) in January 2024 (Rayment & Jarvis, 1997). To account for spatial heterogeneity, for each plot, three to five measurements were taken. To prevent gas diffusion through the soil, a steel collar (external Ø 11.1 cm) was inserted at each sampling point to a depth of 1-5 cm, while ensuring minimal soil disturbance. To standardize the impact of the chamber, the collar was placed into the soil 15-30 minutes before the initial measurement, and a trial was carried out to determine the minimum required waiting time before the initial stabilization of gas exchange processes.

*Soil water infiltration rate*

Measurement was taken at the center of each plot using a metal tube with an inside diameter of 11 cm in March and April 2023. The time required for 100 ml of water to fully infiltrate the soil was recorded, and the procedure was repeated until the infiltration time between each pour stabilized, indicating soil water saturation. The average of the final three infiltration times at saturation was used as the soil water infiltration rate.

*Minimum-, maximum-, and intra-annual variation in soil water content*

Between May 2022 and April 2023, soil moisture was recorded every 15 minutes with a soil moisture logger (TMS-4 datalogger; TOMST s.r.o., Prague, Czech Republic), which measured soil moisture using the electromagnetic signal. Hence, to convert it to volumetric soil water content (m^3^ m^-3^ x 100, i.e. %), HOBO MX Soil Moisture and Temperature Data Logger (Onset Computer, Bourne, MA) was used for the calibration. Then, we calculated the 5^th^ and 95^th^ percentile highest volumetric soil water content as the maximum- and minimum soil water content and the coefficient variation in soil water content within a year as its intra-annual variation. Two sensors in wet forests were broken during the measurement, and therefore these plots were excluded from the analysis of minimum, maximum, and intra-annual variation in soil water content.

*Nutrient mineralization rate*

For each plot, nutrient mineralization rates were estimated by installing resin bags in the soil. Resin bags were prepared by submerging and manually stirring in a 2M KCl solution for 2 hours, followed by drying until 70-80% moisture evaporation (Göransson et al., 2016; Jongen et al., 2021). To capture maximal mineralization rates, resin bags were installed during the wet season (July-August 2023). At each plot, two resin bags were buried at depths of 1 and 8 cm to capture the spatial heterogeneity. After 6 weeks of incubation, resin bags were collected and analyzed at CSIR-Soil Research Institute of Ghana to determine the mineralization rates of ammonium (NH₄^+^), nitrate (NO₃^-^), and phosphate (PO_4_^3-^). We then calculate the average mineralization rates for nitrogen (ammonium and nitrate) and phosphate by averaging the measurements from four bags per plot.

*Nutrient resorption efficiency*

The resorption efficiency of nutrients (N or P) was estimated for each plot as community weighted mean (CWM) of green leaf nutrient concentration minus the litter nutrient concentration per plot, divided by the CWM green leaf nutrient concentration multiplied by 100.

Table S1. Overview of the 16 ecosystem functions with their units, average value (Avg), minimum value (Min), maximum value (Max), the ratio between maximum and minimum value (Ratio), and coefficient of variation (CV).

| Ecosystem function | Unit | Average | Min | Max | Ratio | CV |
| --- | --- | --- | --- | --- | --- | --- |
| Aboveground carbon sequestration | ton ha^-1^ year^-1^ | 4.3 | 1.9 | 10.5 | 5.4 | 44.9 |
| Litter carbon production | ton ha^-1^ year^-1^ | 3.2 | 2.1 | 4.5 | 2.2 | 19.1 |
| Aboveground carbon stock | ton ha^-1^ | 13.0 | 4.4 | 21.6 | 4.9 | 34.0 |
| Belowground carbon stock | ton ha^-1^ | 46.6 | 23.3 | 91.0 | 3.9 | 33.1 |
| Litter decomposition rate | g day^-1^ | 0.024 | 0.0089 | 0.043 | 4.8 | 33.8 |
| Soil respiration | µmol m^-2^ s^-1^ | 2.1 | 0.28 | 7.6 | 27.0 | 72.0 |
| Water infiltration rate | mm hour^-1^ | 27.5 | 4.42 | 69.2 | 15.7 | 70.5 |
| Maximum soil water content (SWC) | % | 27.4 | 10.6 | 51.6 | 4.9 | 41.5 |
| Minimum SWC | % | 14.5 | 3.9 | 32.8 | 8.5 | 48.8 |
| Intra-annual variation in SWC | % | 23.4 | 8.8 | 76.1 | 8.6 | 58.2 |
| Nitrogen (N) resorption rate | % | 55.0 | 39.2 | 72.1 | 1.8 | 14.2 |
| Phosphorus (P) resorption rate | % | 48.8 | 29.8 | 66.6 | 2.2 | 19.0 |
| Litter N flux | ton ha^-1^ year^-1^ | 0.095 | 0.055 | 0.17 | 3.1 | 24.7 |
| Litter P flux | ton ha^-1^ year^-1^ | 0.0055 | 0.0036 | 0.0085 | 2.4 | 23.0 |
| N mineralization | mg kg^-1^ day^-1^ | 6.4 | 0.69 | 17.0 | 24.5 | 65.4 |
| P mineralization | mg kg^-1^ day^-1^ | 0.082 | 0.029 | 0.22 | 7.8 | 47.3 |

Table S2. Results for the 16 best structural equation models (SEMs) for each ecosystem function; a) aboveground carbon sequestration (AGC sequestration, ton ha^-1^ year^-1^), b) litter carbon production (Litter C production, ton ha^-1^ year^-1^), c) aboveground carbon stock (AGC stock, ton ha^-1^), d) belowground carbon stock (BGC stock, ton ha^-1^), e) litter decomposition rate (g day^-1^), f) soil respiration rate (µmol m^-2^ s^-1^), g) soil water infiltration rate (mm h^-1^), h) minimum soil water content (%), i) maximum soil water content (%), j) intra-annual variation in soil water content (%), k) nitrogen resorption rate (N resorption, %), l) phosphorus resorption rate (P resorption, %), m) litter nitrogen flux (Litter N flux, ton ha^-1^ year^-1^), n) litter phosphorus flux (Litter P flux, ton ha^-1^ year^-1^), o) mineralization rate of ammonium and nitrate (N mineralization, mg kg^-1^ day^-1^), and p) mineralization rate of phosphate (P mineralization, mg kg^-1^ day^-1^). (see also Fig. 2). The standardized regression coefficients (Std. coeff), Z-values, and p-values are given for all regressions, and the R^2^ of the endogenous variables (i.e. variables that are affected by other variables: measures of forest attributes and ecosystem functions). All 16 models were accepted (p = 0.085, 0.17, 0.060, 0.054, 0.71, 0.14, 0.30, 0.81, 0.16, 0.20, 0.14, 0.06, 0.32, 0.14, 0.073, 0.25; and χ2 = 6.6, 5.1, 7.4, 7.6, 1.4, 5.4, 3.7, 0.97, 5.2, 4.6, 5.6, 7.4, 3.5, 5.4, 7.0, 4.1, respectively). For abbreviations, see the captions in Figure S4.

| **﻿Response variable** | **﻿Predictor variable** | **﻿Std. coeff** | **﻿Z- value** | **﻿p-value** |
| --- | --- | --- | --- | --- |
| a) AGC sequestration | Climatic wetness  Clay content  Stand basal area  Species richness  CWM wood density | 0.020  -0.21  0.47  -0.24  -0.46 | 0.11  -1.8  3.5  -2.4  -3.5 | 0.91  0.078  <0.001  0.016  0.001 |
| Stand basal area | Climatic wetness  Clay content | 0.71  0.011 | 5.1  0.081 | <0.001  0.94 |
| Species richness | Climatic wetness  Clay content | -0.25  0.41 | -1.4  2.2 | 0.17  0.028 |
| CWM wood density | Climatic wetness  Clay content | -0.80  0.16 | -5.9  1.2 | <0.001  0.23 |
| R^2^ AGC sequestration | 0.69 |  |  |  |
| R^2^ Stand basal area | 0.51 |  |  |  |
| R^2^ Species richness | 0.12 |  |  |  |
| R^2^ CWM wood density | 0.52 |  |  |  |
| b) Litter C production | Climatic wetness  Soil P  Tree density  Species richness  CWM LMA | 0.28  0.21  0.46  0.19  0.10 | 2.1  1.9  3.4  1.7  0.9 | 0.036  0.064  0.001  0.098  0.36 |
| Tree density | Climatic wetness  Soil P | 0.56  0.086 | 4.1  0.63 | <0.001  0.53 |
| Species richness | Climatic wetness  Soil P | -0.057  0.20 | -0.35  1.2 | 0.73  0.23 |
| CWM LMA | Climatic wetness  Soil P | -0.091  -0.14 | -0.55  -0.87 | 0.58  0.38 |
| R^2^ Litter C production | 0.56 |  |  |  |
| R^2^ Tree density | 0.33 |  |  |  |
| R^2^ Species richness | 0.040 |  |  |  |
| R^2^ CWM LMA | 0.032 |  |  |  |
| c) AGC stock | Climatic wetness  Soil bulk density  Stand basal area  Species richness  CWM WD | -0.081  -0.031  1.1  0.045  0.22 | -2.6  -1.7  44.9  2.5  8.5 | 0.01  0.10  <0.001  0.012  <0.001 |
| Stand basal area | Climatic wetness  Soil bulk density | 0.71  -0.12 | 6.1  -1.0 | <0.001  0.30 |
| Species richness | Climatic wetness  Soil bulk density | -0.044  -0.14 | -0.27  -0.82 | 0.79  0.41 |
| CWM WD | Climatic wetness  Soil bulk density | -0.73  -0.22 | -6.5  -2.0 | <0.001  0.82 |
| R^2^ AGC stock | 0.99 |  |  |  |
| R^2^ Stand basal area | 0.53 |  |  |  |
| R^2^ Species richness | 0.019 |  |  |  |
| R^2^ CWM WD | 0.55 |  |  |  |
| d) BGC stock | Climatic wetness  Soil bulk density  Stand basal area  Species richness  CWM leaf N | 0.56  0.66  -0.13  0.55  0.31 | 2.2  6.3  -0.86  5.4  1.3 | 0.031  <0.001  0.39  <0.001  0.19 |
| Stand basal area | Climatic wetness  Soil bulk density | 0.71  -0.12 | 6.1  -1.0 | <0.001  0.30 |
| Species richness | Climatic wetness  Soil bulk density | -0.044  -0.14 | -0.27  -0.82 | 0.79  0.41 |
| CWM leaf N | Climatic wetness  Soil bulk density | -0.90  0.028 | -12.7  0.39 | <0.001  0.70 |
| R^2^ BGC stock | 0.65 |  |  |  |
| R^2^ Stand basal area | 0.53 |  |  |  |
| R^2^ Species richness | 0.019 |  |  |  |
| R^2^ CWM leaf N | 0.82 |  |  |  |
| e) Litter decomposition rate | Climatic wetness  Soil P  Stand basal area  Species diversity  CWM leaf N | 0.44  0.41  -0.49  -0.018  0.55 | 1.3  3.1  -2.6  -0.12  1.8 | 00.19  0.002  0.010  0.90  0.074 |
| Stand basal area | Climatic wetness  Soil P | 0.70  0.095 | 6.1  0.82 | <0.001  0.41 |
| Species diversity | Climatic wetness  Soil P | -0.45  -0.076 | -3.1  -0.51 | 0.002  0.61 |
| CWM leaf N | Climatic wetness  Soil P | -0.90  -0.021 | -12.7  -0.29 | <0.001  0.77 |
| R^2^ Litter decomposition rate | 0.40 |  |  |  |
| R^2^ Stand basal area | 0.52 |  |  |  |
| R^2^ Species diversity | 0.22 |  |  |  |
| R^2^ CWM leaf N | 0.82 |  |  |  |
| f) Soil respiration rate | Climatic wetness  Soil bulk density  Tree density  Species richness  CWM wood density | 0.48  0.24  -0.47  0.60  0.042 | 2.3  1.7  -0.29  4.5  0.21 | 0.024  0.085  0.004  <0.001  0.83 |
| Tree density | Climatic wetness  Soil bulk density | 0.59  0.17 | 4.4  1.2 | <0.001  0.22 |
| Species richness | Climatic wetness  Soil bulk density | -0.044  0.14 | -0.27  -0.82 | 0.79  0.41 |
| CWM wood density | Climatic wetness  Soil bulk density | -0.73  -0.22 | -6.5  -2.0 | <0.001  0.74 |
| R^2^ Soil respiration rate | 0.46 |  |  |  |
| R^2^ Tree density | 0.35 |  |  |  |
| R^2^ Species richness | 0.019 |  |  |  |
| R^2^ CWM wood density | 0.55 |  |  |  |
| g) Soil water infiltration rate | Climatic wetness  Soil bulk density  Stand basal area  Species evenness  CWM WD | -0.085  0.34  -0.23  -0.092  -0.15 | -0.30  -2.0  -1.0  -0.53  -0.64 | 0.77  0.047  0.31  0.60  0.52 |
| Stand basal area | Climatic wetness  Soil bulk density | 0.71  -0.12 | 6.1  -1.0 | <0.001  0.30 |
| Species evenness | Climatic wetness  Soil bulk density | -0.36  0.23 | -2.4  1.5 | 0.016  0.13 |
| CWM WD | Climatic wetness  Soil bulk density | -0.73  -0.22 | -6.5  -2.0 | <0.001  0.048 |
| R^2^ Soil water infiltration rate | 0.14 |  |  |  |
| R^2^ Stand basal area | 0.53 |  |  |  |
| R^2^ Species evenness | 0.20 |  |  |  |
| R^2^ CWM WD | 0.55 |  |  |  |
| h) Minimum soil water content | Climatic wetness  Soil clay content  Stand basal area  Species diversity  CWM leaf N | 0.66  -0.25  -0.17  0.10  -0.50 | 2.7  -2.1  -1.4  1.0  -2.0 | 0.006  0.034  0.17  0.30  0.043 |
| Stand basal area | Climatic wetness  Soil clay content | 0.72  -0.020 | 5.0  -0.14 | <0.001  0.89 |
| Species diversity | Climatic wetness  Soil clay content | -0.40  -0.12 | -2.2  -0.66 | 0.026  0.51 |
| CWM leaf N | Climatic wetness  Soil clay content | -0.79  -0.24 | -11.1  -3.4 | <0.001  0.001 |
| R^2^ Minimum soil water content | 0.75 |  |  |  |
| R^2^ Stand basal area | 0.50 |  |  |  |
| R^2^ Species diversity | 0.22 |  |  |  |
| R^2^ CWM leaf N | 0.88 |  |  |  |

| i) Maximum soil water content | Climatic wetness  Soil clay content  Tree density  Species richness  CWM leaf N | 0.54  -0.21  0.14  -0.043  -0.33 | 2.2  -1.6  1.3  -0.43  -1.2 | 0.031  0.12  0.21  0.67  0.23 | |
| --- | --- | --- | --- | --- | --- |
| Tree density | Climatic wetness  Soil clay content | 0.54  0.017 | 3.2  0.098 | 0.002  0.92 | |
| Species richness | Climatic wetness  Soil clay content | -0.25  0.39 | -1.3  2.0 | 0.19  0.042 |  |
| CWM leaf N | Climatic wetness  Soil clay content | -0.79  -0. 24 | -11.1  -3.4 | <0.001  0.001 | |
| R^2^ Maximum soil water content | 0.70 |  |  |  | |
| R^2^ Tree density | 0.30 |  |  |  | |
| R^2^ Species richness | 0.11 |  |  |  | |
| R^2^ CWM leaf N | 0.88 |  |  |  | |
| j) Intra-annual variation in soil water content | Climatic wetness  Soil TEB  Stand basal area  Species diversity  CWM LMA | -0.93  -0.65  0.009  -0.53  0.23 | -3.9  -3.7  0.051  -3.6  1.7 | <0.001  <0.001  0.96  <0.001  0.082 | |
| Stand basal area | Climatic wetness  Soil TEB | 0.76  0.071 | 4.6  0.43 | <0.001  0.67 | |
| Species diversity | Climatic wetness  Soil TEB | -0.57  -0.16 | -2.8  -0.79 | 0.006  0.43 | |
| CWM LMA | Climatic wetness  Soil TEB | -0.18  -0.008 | -0.76  -0.036 | 0.45  0.97 | |
| R^2^ Intra-annual variation in soil water content | 0.48 |  |  |  | |
| R^2^ Stand basal area | 0.50 |  |  |  | |
| R^2^ Species diversity | 0.23 |  |  |  | |
| R^2^ CWM LMA | 0.029 |  |  |  | |
| k) N resorption | Climatic wetness  Soil N  Stand basal area  Species evenness  CWM LMA | 0.72  0.25  -0.12  0.23  -0.34 | 4.3  1.9  -0.73  1.8  -2.9 | <0.001  0.064  0.47  0.072  0.004 | |
| Stand basal area | Climatic wetness  Soil N | 0.67  0.18 | 5.7  1.6 | <0.001  0.12 | |
| Species evenness | Climatic wetness  Soil N | -0.29  -0.33 | -2.0  -2.2 | 0.052  0.026 | |
| CWM LMA | Climatic wetness  Soil N | -0.18  0.27 | -1.1  1.6 | 0.28  0.11 | |
| R^2^ N resorption | 0.56 |  |  |  | |
| R^2^ Stand basal area | 0.54 |  |  |  | |
| R^2^ Species evenness | 0.25 |  |  |  | |
| R^2^ CWM LMA | 0.077 |  |  |  | |
| l) P resorption | Climatic wetness  Soil bulk density  Stand basal area  Species richness  CWM wood density | 0.52  0.42  0.13  0.31  0.29 | 2.2  3.0  0.70  2.3  1.5 | 0.027  0.003  0.49  0.020  0.14 | |
| Stand basal area | Climatic wetness  Soil bulk density | 0.71  -0.12 | 6.1  -1.0 | <0.001  0.30 | |
| Species richness | Climatic wetness  Soil bulk density | -0.044  -0.14 | -0.27  -0.82 | 0.79  0.41 | |
| CWM wood density | Climatic wetness  Soil bulk density | -0.73  -0.22 | -6.5  -2.0 | <0.001  0.048 | |
| R^2^ P resorption | 0.37 |  |  |  | |
| R^2^ Stand basal area | 0.53 |  |  |  | |
| R^2^ Species richness | 0.019 |  |  |  | |
| R^2^ CWM wood density | 0.55 |  |  |  | |
| m) Litter N flux | Climatic wetness  Soil sand content  Stand basal area  Species evenness  CWM LMA | -0.73  0.34  0.40  -0.22  0.19 | -3.7  2.0  2.7  -1.8  1.7 | <0.001  0.049  0.007  0.073  0.087 | |
| Stand basal area | Climatic wetness  Soil sand content | 0.83  0.15 | 4.9  0.88 | <0.001  0.38 | |
| Species evenness | Climatic wetness  Soil sand content | -0.017  0.50 | -0.080  2.4 | 0.94  0.018 | |
| CWM LMA | Climatic wetness  Soil sand content | 0.18  0.39 | 0.45  0.098 | 0.18  0.39 | |
| R^2^ Litter N flux | 0.60 |  |  |  | |
| R^2^ Stand basal area | 0.52 |  |  |  | |
| R^2^ Species evenness | 0.26 |  |  |  | |
| R^2^ CWM LMA | 0.082 |  |  |  | |
| n) Litter P flux | Climatic wetness  Soil bulk density  Tree density  Species richness  CWM wood density | -0.85  -0.31  0.34  -0.12  -0.15 | -3.9  -2.2  2.1  -0.89  -0.77 | <0.001  0.028  0.039  0.37  0.44 | |
| Tree density | Climatic wetness  Soil bulk density | 0.59  0.17 | 4.4  1.2 | <0.001  0.22 | |
| Species richness | Climatic wetness  Soil bulk density | -0.044  -0.14 | -0.27  -0.82 | 0.79  0.41 | |
| CWM wood density | Climatic wetness  Soil bulk density | -0.73  -0.22 | -6.5  -2.0 | <0.001  0.048 | |
| R^2^ Litter P flux | 0.40 |  |  |  | |
| R^2^ Tree density | 0.35 |  |  |  | |
| R^2^ Species richness | 0.019 |  |  |  | |
| R^2^ CWM wood density | 0.55 |  |  |  | |
| o) N mineralization | Climatic wetness  Soil N  Tree density  Species evenness  CWM leaf N | -0.47  -0.40  0.54  0.16  -0.27 | -1.5  -2.8  3.5  1.1  -0.88 | 0.15  0.005  0.001  0.29  0.38 | |
| Tree density | Climatic wetness  Soil N | 0.56  0.034 | 4.0  0.24 | <0.001  0.81 | |
| Species evenness | Climatic wetness  Soil N | -0.29  -0.33 | -2.0  -2.2 | 0.052  0.026 | |
| CWM leaf N | Climatic wetness  Soil N | -0.92  0.049 | -12.6  0.67 | <0.001  0.50 | |
| R^2^ N mineralization | 0.42 |  |  |  | |
| R^2^ Tree density | 0.33 |  |  |  | |
| R^2^ Species evenness | 0.25 |  |  |  | |
| R^2^ CWM leaf N | 0.82 |  |  |  | |
| p) P mineralization | Climatic wetness  Soil clay content  Tree density  Species richness  CWM leaf N | -0.74  0.39  -0.42  0.12  -0.54 | -2.3  2.2  -2.7  0.92  -1.6 | 0.020  0.031  0.007  0.36  0.12 | |
| Tree density | Climatic wetness  Soil clay content | 0.55  0.042 | 3.4  0.26 | 0.001  0.79 | |
| Species richness | Climatic wetness  Soil clay content | -0.25  0.41 | -1.4  2.2 | 0.17  0.028 | |
| CWM leaf N | Climatic wetness  Soil clay content | -0.77  -0.25 | -10.5  -3.4 | <0.001  0.001 | |
| R^2^ P mineralization | 0.45 |  |  |  | |
| R^2^ Tree density | 0.33 |  |  |  | |
| R^2^ Species richness | 0.12 |  |  |  | |
| R^2^ CWM leaf N | 0.86 |  |  |  | |

Table S3. Results of the top three structural equation models of a) aboveground carbon sequestration (AGC sequestration), b) litter carbon production (Litter C production), c) aboveground carbon stock (AGC stock), d) belowground carbon stock (BGC stock), e) litter decomposition rate, f) soil respiration rate, g) soil water infiltration rate, h) minimum soil water content (SWC), i) maximum SWC, j) intra-annual variation in SWC, k) nitrogen resorption rate (N resorption), l) phosphorus resorption rate (P resorption), m) litter nitrogen flux (Litter N flux), n) litter phosphorus flux (Litter P flux), o) mineralization rate of ammonium and nitrate (N mineralization), and p) mineralization rate of phosphate (P mineralization). The degree of freedom (DF), the test statistic (minimum function chi-square), the model p-value, the model Akaike Information Criterion (AIC), and the variance of ecosystem functions explained by the model (R^2^) are given for each model. Abbreviations are as follows; soil clay content (Clay), soil sand content (Sand), soil bulk density (BD), soil phosphorus (P), soil total nitrogen (TN), soil total exchangeable bases (TEB), stand basal area (Stand BA), tree density (Density), and Community weighted mean (CWM) of wood density (WD), leaf nitrogen (leaf N), or leaf mass per area (LMA).

| **﻿Ecosystem functions** | **﻿Soil conditions** | **﻿Forest structure** | **﻿Taxonomic diversity** | **Functional composition** | **DF** | **χ2** | **p-value** | **AIC** | **﻿R^2^** |
| --- | --- | --- | --- | --- | --- | --- | --- | --- | --- |
| a) AGC sequestration | Clay  TN  TEB | Stand BA  Stand BA  Stand BA | Richness  Richness  Richness | CWM WD  CWM WD  CWM WD | 3  3  3 | 6.6  5.0  5.0 | 0.085  0.17  0.10 | 335.1  329.8  336.9 | 0.692  0.687  0.685 |
| b) Litter C production | Phosphorus  Phosphorus  Phosphorus | Density  Density  Density | Richness  Richness  Richness | CWM LMA  CWM leaf N  CWM WD | 3  3  3 | 5.1  5.2  3.2 | 0.17  0.16  0.37 | 387.3  326.7  363.0 | 0.564  0.546  0.545 |
| c) AGC stock | BD  Phosphorus  Sand | Stand BA  Stand BA  Stand BA | Richness  Richness  Richness | CWM WD  CWM WD  CWM WD | 3  3  3 | 7.4  6.4  6.9 | 0.060  0.093  0.075 | 215.7  219.8  212.3 | 0.9894  0.9890  0.9887 |
| d) BGC stock | BD  BD  BD | Stand BA  Density  Stand BA | Richness  Richness  Richness | CWM leaf N  CWM WD  CWM WD | 3  3  3 | 7.7  6.3  7.4 | 0.054  0.097  0.060 | 309.4  321.2  343.7 | 0.653  0.636  0.623 |
| e) Decomposition rates | Phosphorus  Phosphorus  Phosphorus | Stand BA  Stand BA  Stand BA | Diversity  Richness  Evenness | CWM leaf N  CWM leaf N  CWM leaf N | 3  3  3 | 1.4  7.5  4.8 | 0.71  0.057  0.19 | 319.7  327.1  322.0 | 0.402  0.399  0.395 |
| f) Soil respiration | BD  BD  BD | Density  Density  Density | Richness  Richness  Richness | CWM WD  CWM LMA  CWM leaf N | 3  3  3 | 5.4  7.1  6.3 | 0.14  0.070  0.097 | 371.6  399.6  338.5 | 0.4591  0.4590  0.4577 |
| g) Soil water infiltration rate | BD  BD  BD | Stand BA  Stand BA  Stand BA | Evenness  Diversity  Richness | CWM WD  CWM WD  CWM WD | 3  3  3 | 3.7  2.7  7.4 | 0.29  0.45  0.060 | 366.0  364.3  373.4 | 0.136  0.135  0.133 |
| h) Minimum SWC | Clay  TEB  Clay | Stand BA  Stand BA  Stand BA | Diversity  Evenness  Richness | CWM leaf N  CWM leaf N  CWM leaf N | 3  3  3 | 0.97  6.0  5.5 | 0.81  0.11  0.14 | 261.3  270.8  266.8 | 0.745  0.741  0.738 |
| i) Maximum SWC | Clay  Clay  Clay | Density  Density  Density | Richness  Diversity  Evenness | CWM leaf N  CWM leaf N  CWM leaf N | 3  3  3 | 5.2  4.6  4.9 | 0.16  0.20  0.18 | 283.6  279.1  279.2 | 0.695  0.693  0.692 |
| j) Intra-annual variation in SWC | TEB  TEB  TEB | Stand BA  Stand BA  Density | Diversity  Evenness  Evenness | CWM LMA  CWM leaf N  CWM leaf N | 3  3  3 | 4.6  6.0  7.5 | 0.20  0.11  0.059 | 358.3  297.7  309.4 | 0.479  0.473  0.467 |
| k) N resorption | TN  TEB  Clay | Stand BA  Stand BA  Density | Evenness  Evenness  Diversity | CWM LMA  CWM LMA  CWM LMA | 3  3  3 | 5.6  5.8  7.0 | 0.14  0.12  0.072 | 365.1  382.6  380.9 | 0.560  0.538  0.530 |
| l) P resorption | BD  BD  BD | Stand BA  Density  Stand BA | Richness  Richness  Richness | CWM WD  CWM WD  CWM LMA | 3  3  3 | 7.4  5.4  6.2 | 0.060  0.14  0.10 | 360.8  372.3  389.1 | 0.368  0.361  0.360 |
| m) Litter N flux | Sand  Sand  Sand | Stand BA  Density  Stand BA | Evenness  Evenness  Evenness | CWM LMA  CWM LMA  CWM WD | 3  3  3 | 3.5  4.6  2.8 | 0.32  0.21  0.43 | 359.8  375.2  333.4 | 0.604  0.591  0.586 |
| n) Litter P flux | BD  BD  TN | Density  Density  Density | Richness  Richness  Richness | CWM WD  CWM LMA  CWM LMA | 3  3  3 | 5.4  7.1  5.2 | 0.14  0.070  0.16 | 372.0  400.1  377.4 | 0.3984  0.3979  0.3889 |
| o) N mineralization | TN  TN  TN | Density  Density  Density | Evenness  Richness  Evenness | CWM leaf N  CWM leaf N  CWM WD | 3  3  3 | 7.0  7.7  3.0 | 0.073  0.052  0.39 | 329.1  331.1  366.5 | 0.415  0.410  0.406 |
| p) P mineralization | Clay  Clay  Clay | Density  Density  Density | Richness  Diversity  Diversity | CWM leaf N  CWM leaf N  CWM LMA | 3  3  3 | 4.1  3.1  7.0 | 0.25  0.38  0.072 | 324.1  318.9  388.5 | 0.449  0.442  0.435 |

Table S4. Absolute weighted-average standardized effects of each predictor variable—environmental conditions (climatic wetness and soil conditions) and forest attributes (forest structure, taxonomic diversity, and functional composition) of a) aboveground carbon sequestration (AGC sequestration), b) litter carbon production (Litter C production), c) aboveground carbon stock (AGC stock), d) belowground carbon stock (BGC stock), e) litter decomposition rate, f) soil respiration rate, g) soil water infiltration rate, h) minimum soil water content (SWC), i) maximum SWC, j) intra-annual variation in SWC, k) nitrogen resorption rate (N resorption), l) phosphorus resorption rate (P resorption), m) litter nitrogen flux (Litter N flux), n) litter phosphorus flux (Litter P flux), o) mineralization rate of ammonium and nitrate (N mineralization), and p) mineralization rate of phosphate (P mineralization). The number of selected models based on R^2^ of the final response variable (N) is given for each ecosystem function.

| **﻿Ecosystem functions** | **﻿Climatic wetness** | **﻿Soil conditions** | **﻿Forest structure** | **﻿Taxonomic diversity** | **Functional composition** |  | **﻿N** |
| --- | --- | --- | --- | --- | --- | --- | --- |
| a) AGC sequestration | 0.64 | 0.23 | 0.43 | 0.25 | 0.38 |  | 11 |
| b) Litter C production | 0.52 | 0.27 | 0.46 | 0.12 | 0.16 |  | 6 |
| c) AGC stock | 0.58 | 0.14 | 1.1 | 0.034 | 0.13 |  | 52 |
| d) BGC stock | 0.17 | 0.60 | 0.085 | 0.53 | 0.20 |  | 3 |
| e) Decomposition rates | 0.39 | 0.36 | 0.45 | 0.081 | 0.45 |  | 4 |
| f) Soil respiration | 0.15 | 0.076 | 0.48 | 0.59 | 0.085 |  | 3 |
| g) Soil water infiltration rate | 0.11 | 0.30 | 0.22 | 0.094 | 0.13 |  | 4 |
| h) Minimum SWC | 0.87 | 0.12 | 0.15 | 0.096 | 0.21 |  | 60 |
| i) Maximum SWC | 0.83 | 0.10 | 0.14 | 0.034 | 0.085 |  | 74 |
| j) Intra-annual variation in SWC | 0.66 | 0.56 | 0.024 | 0.50 | 0.55 |  | 3 |
| k) N resorption | 0.68 | 0.077 | 0.15 | 0.22 | 0.29 |  | 2 |
| l) P resorption | 0.39 | 0.30 | 0.10 | 0.31 | 0.22 |  | 4 |
| m) Litter N flux | 0.40 | 0.32 | 0.41 | 0.16 | 0.19 |  | 6 |
| n) Litter P flux | 0.48 | 0.22 | 0.28 | 0.16 | 0.17 |  | 5 |
| o) N mineralization | 0.034 | 0.45 | 0.55 | 0.11 | 0.14 |  | 6 |
| p) P mineralization | 0.58 | 0.56 | 0.38 | 0.15 | 0.41 |  | 5 |

Table S5. Overview of the six soil physical and chemical properties with their units, average value (Avg), minimum value (Min), maximum value (Max), the ratio between maximum and minimum value (Ratio), and coefficient of variation (CV).

| Soil property | Unit | Average | Min | Max | Ratio | CV |
| --- | --- | --- | --- | --- | --- | --- |
| Soil bulk density | g cm^-3^ | 1.2 | 0.59 | 1.7 | 2.8 | 25.1 |
| Soil sand content | % | 54.5 | 30.0 | 86.0 | 2.9 | 30.3 |
| Soil clay content | % | 16.8 | 4.0 | 34.0 | 8.5 | 50.9 |
| Soil total nitrogen | mg g^-1^ | 2.4 | 0.96 | 3.5 | 3.6 | 25.0 |
| Soil available phosphorus | µg g^-1^ | 3.8 | 1.2 | 13.5 | 10.9 | 67.3 |
| Soil total exchangeable bases | meq 100g^-1^ | 7.2 | 2.4 | 17.5 | 7.2 | 58.3 |

Table S6. Overview of the eight forest attributes with their units, average value (Avg), minimum value (Min), maximum value (Max), the ratio between maximum and minimum value (Ratio), and coefficient of variation (CV).

| Forest attribute | Unit | Average | Min | Max | Ratio | CV |
| --- | --- | --- | --- | --- | --- | --- |
| Stand basal area | m^-2^ ha^-1^ | 11.5 | 3.2 | 21.3 | 6.7 | 38.3 |
| Tree density | # ha^-1^ | 7370 | 3330 | 14450 | 4.3 | 35.6 |
| Species richness | # 625m^-2^ | 33.1 | 20.0 | 51.0 | 2.6 | 23.9 |
| Species evenness | Unitless | 0.35 | 0.13 | 0.95 | 7.5 | 49.0 |
| Species diversity | Unitless | 10.8 | 4.64 | 19.0 | 4.1 | 35.0 |
| Community weighted mean (CWM) leaf nitrogen content | mg g^-1^ | 31.9 | 25.1 | 39.4 | 1.6 | 14.7 |
| CWM leaf mass per area | g cm^-2^ | 60.3 | 50.6 | 74.6 | 1.5 | 9.6 |
| CWM wood density | g cm^-3^ | 0.45 | 0.33 | 0.57 | 1.7 | 11.0 |


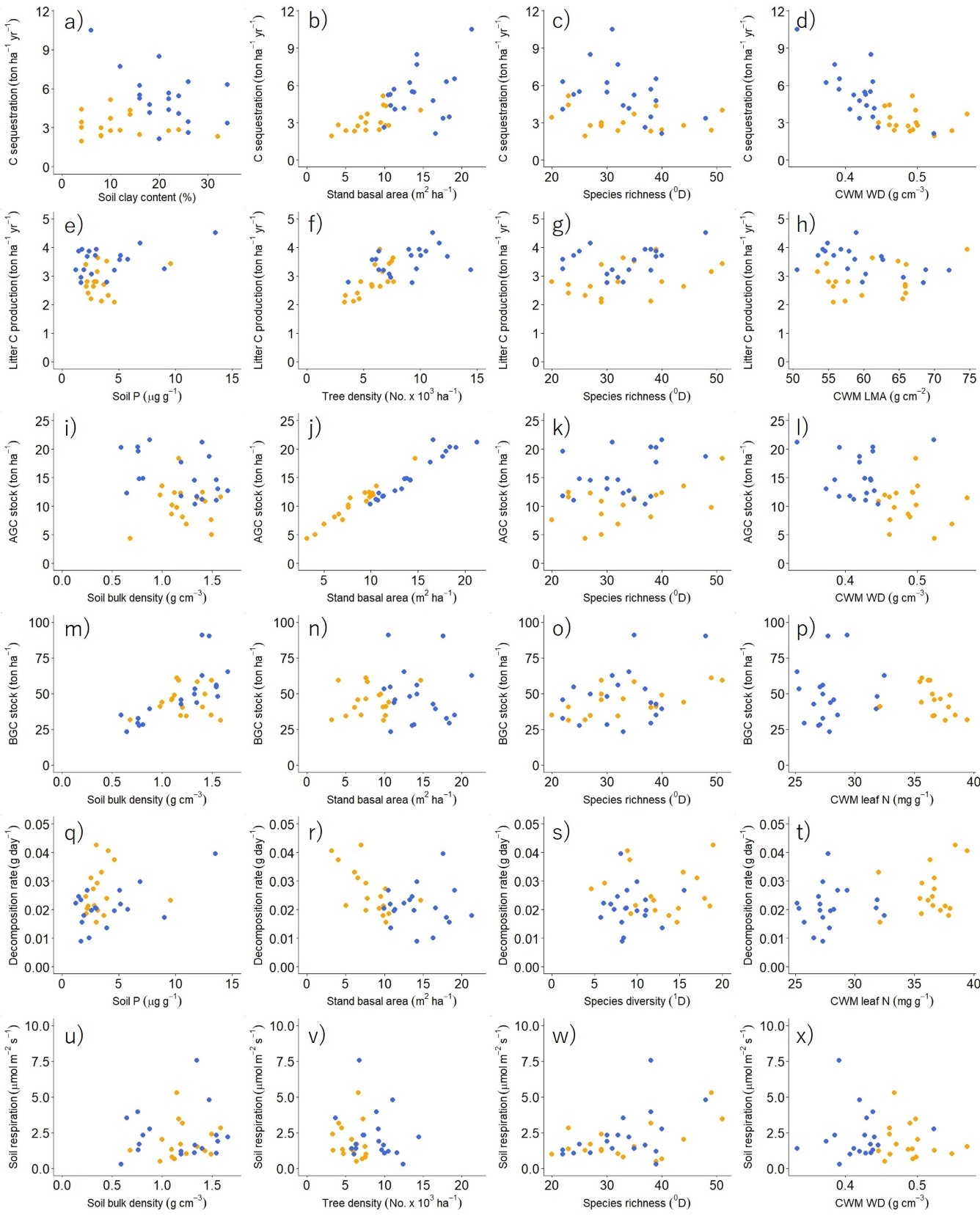


Figure S1. Bivariate relationship between carbon functions [aboveground carbon (C) sequestration, litter C production, aboveground carbon (AGC) stock, belowground carbon (BGC) stock, litter decomposition rate, and soil respiration] and soil properties [soil clay content, soil phosphorus (P), or soil bulk density], forest structure (stand basal area or tree density), diversity (species richness or species diversity based on Hill number), and functional composition [community-weighted mean (CWM) of wood density (WD), leaf mass per area (LMA), or leaf nitrogen concentration (leaf N)]. Data are shown for secondary tropical dry forest plots (orange, N=17) and wet forest plots (blue, N=19). The chosen variables of environmental conditions and forest attributes were the ones selected in the best model in the structural equation models (Fig. 2). Note that these bivariate relationships are for illustration purposes only and may not necessarily provide the same results as in the structural equation models.


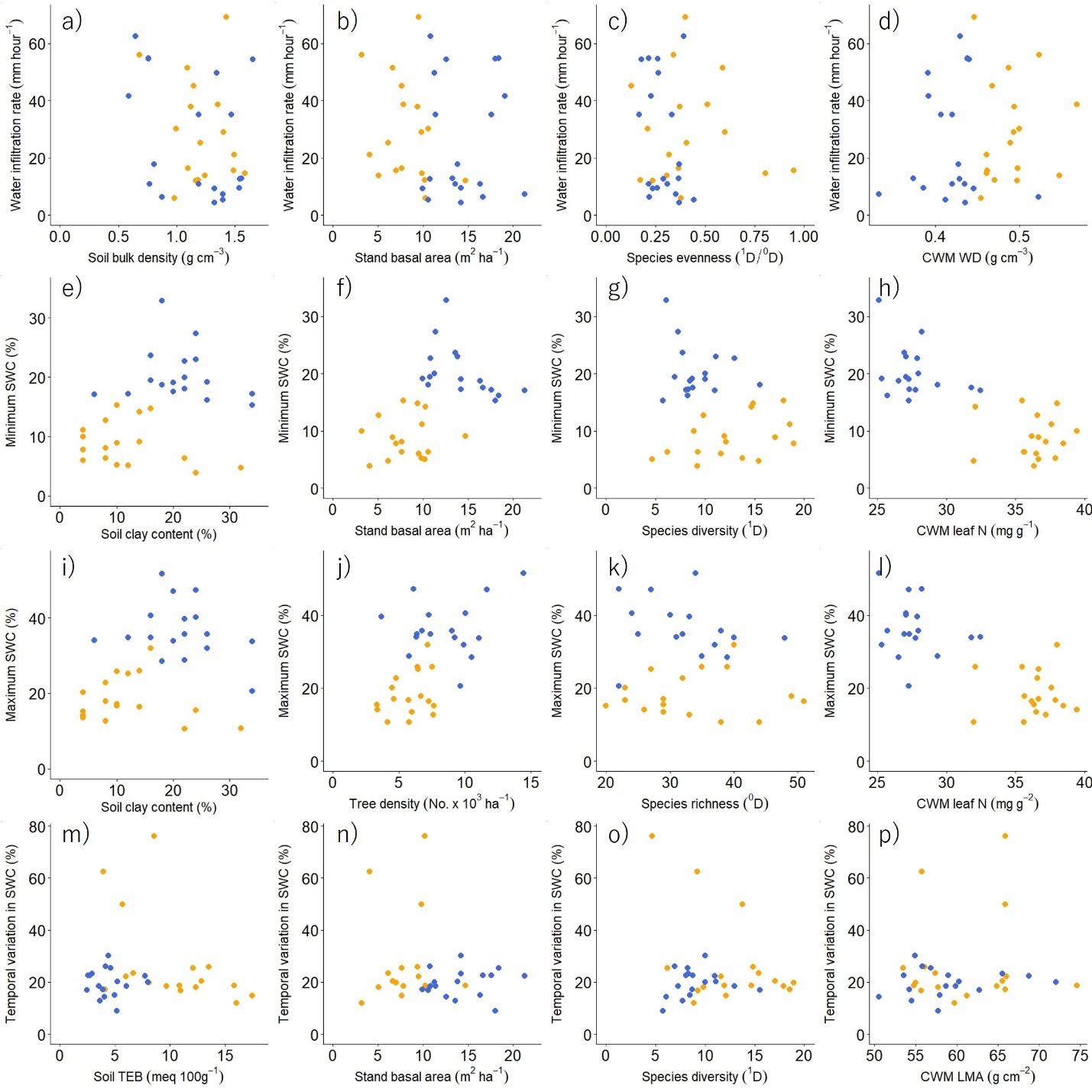


Figure S2. Bivariate relationship between water functions [water infiltration rate, and maximum-, minimum-, and intra-annual variation in soil water content (SWC) in a year] and soil properties [soil bulk density, soil clay content, or soil total exchangeable bases (TEB)], forest structure (stand basal area or tree density), taxonomic diversity (species richness, species evenness, or species diversity based on Hill numbers), and functional composition [community-weighted mean (CWM) of wood density (WD), leaf nitrogen (leaf N), or leaf mass per area (LMA)]. Data are shown for secondary tropical dry forest plots (orange, N=17) and wet forest plots (blue, N=19 for soil water infiltration rate and N=17 for the others). The chosen variables of environmental conditions and forest attributes were the ones selected in the best model in the structural equation models (Fig. 2). Note that these bivariate relationships are for illustration purposes only and may not necessarily provide the same results as in the structural equation models.


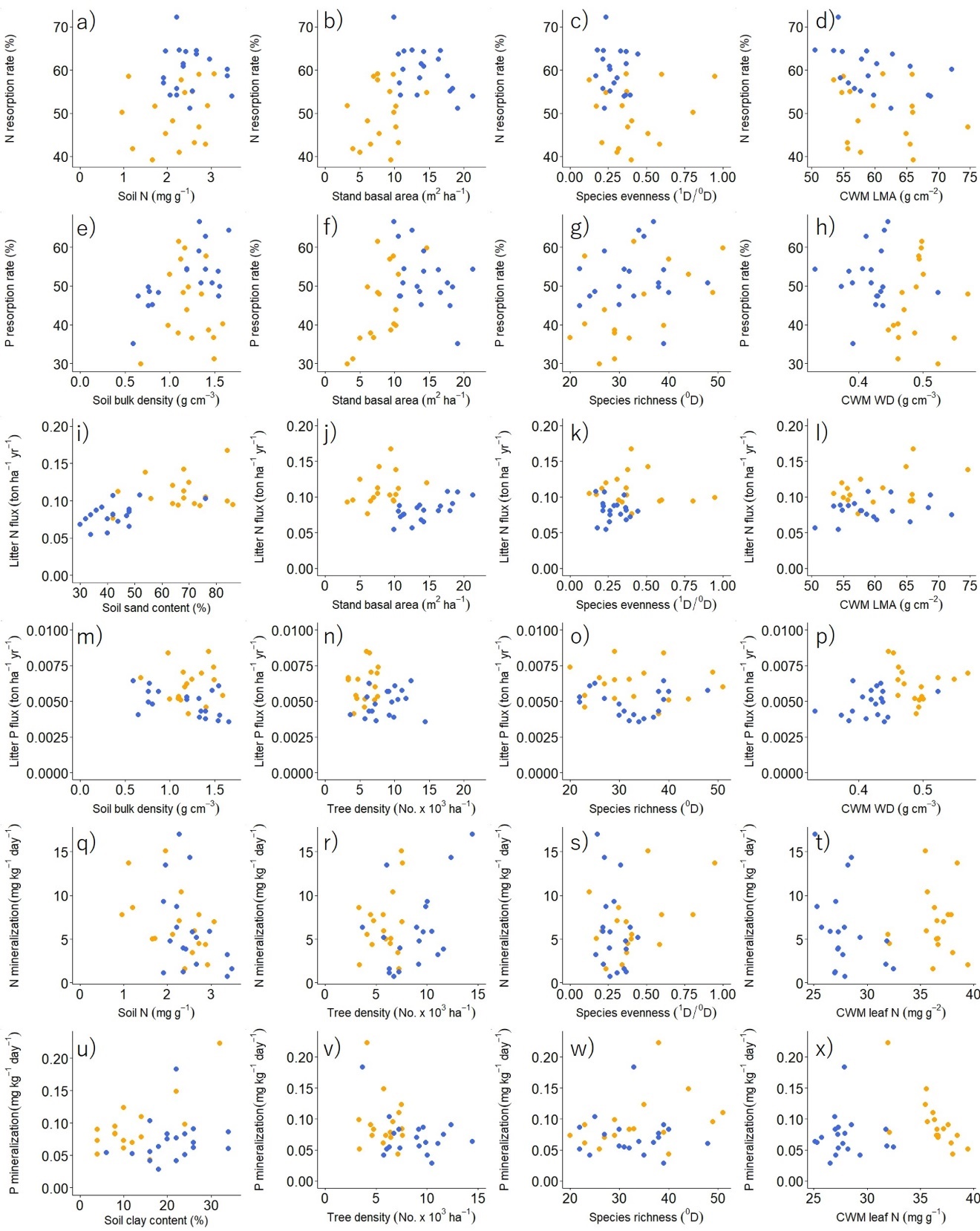


Figure S3. Bivariate relationship between nutrient functions [nitrogen (N) or phosphorus (P) resorption rate, litter N or P flux, and mineralization rates of N or P] and soil properties (soil N, soil bulk density, and soil sand or clay content), forest structure (stand basal area or tree density), taxonomic diversity (species richness or species evenness based on Hill numbers), and functional composition [community-weighted mean (CWM) of wood density (WD), leaf mass per area (LMA), and leaf nitrogen concentration (leaf N)]. Data are shown for secondary tropical dry forest plots (orange, N=17) and wet forest plots (blue, N=19). The chosen variables of environmental conditions and forest attributes were the ones selected in the best model in the structural equation models (Fig. 2). Note that these bivariate relationships are for illustration purposes only and may not necessarily provide the same results as in the structural equation models.


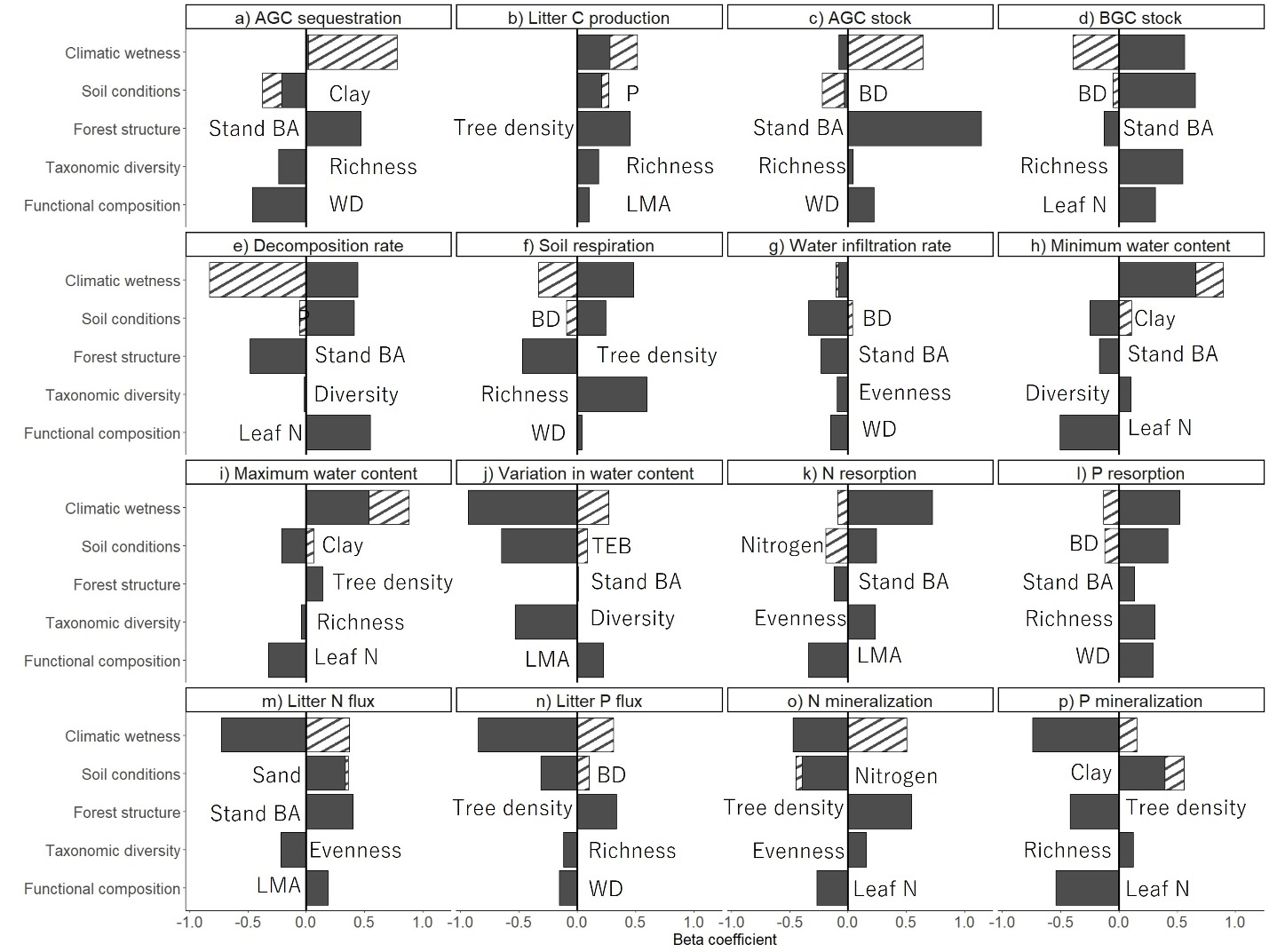


Figure S4. Beta coefficients of environmental conditions (climatic wetness and soil conditions) and forest attributes (forest structure, taxonomic diversity, and functional composition) on 16 different ecosystem functions: a) aboveground carbon sequestration (AGC sequestration, ton ha^-1^ year^-1^), b) litter carbon production (Litter C production, ton ha^-1^ year^-1^), c) aboveground carbon stock (AGC stock, ton ha^-1^), d) belowground carbon stock (BGC stock, ton ha^-1^), e) litter decomposition rate (g day^-1^), f) soil respiration rate (µmol m^-2^ s^-1^), g) soil water infiltration rate (mm h^-1^), h) minimum soil water content (%), i) maximum soil water content (%), j) intra-annual variation in soil water content (%), k) nitrogen resorption rate (N resorption, %), l) phosphorus resorption rate (P resorption, %), m) litter nitrogen flux (Litter N flux, ton ha^-1^ year^-1^), n) litter phosphorus flux (Litter P flux, ton ha^-1^ year^-1^), o) mineralization rate of ammonium and nitrate (N mineralization, mg kg^-1^ day^-1^), and p) mineralization rate of phosphate (P mineralization, mg kg^-1^ day^-1^) based on the best models in structural equation models (Fig. 2). The filled bars show the direct effects of environmental conditions and forest attributes, and the hatched bars show the indirect effects of environmental conditions. Abbreviations are as follows; soil clay content (Clay), soil sand content (Sand), soil bulk density (BD), soil phosphorus (P), soil nitrogen (Nitrogen), soil total exchangeable bases (TEB), stand basal area (Stand BA), wood density (WD), leaf mass per area (LMA), and leaf nitrogen concentration (Leaf N).


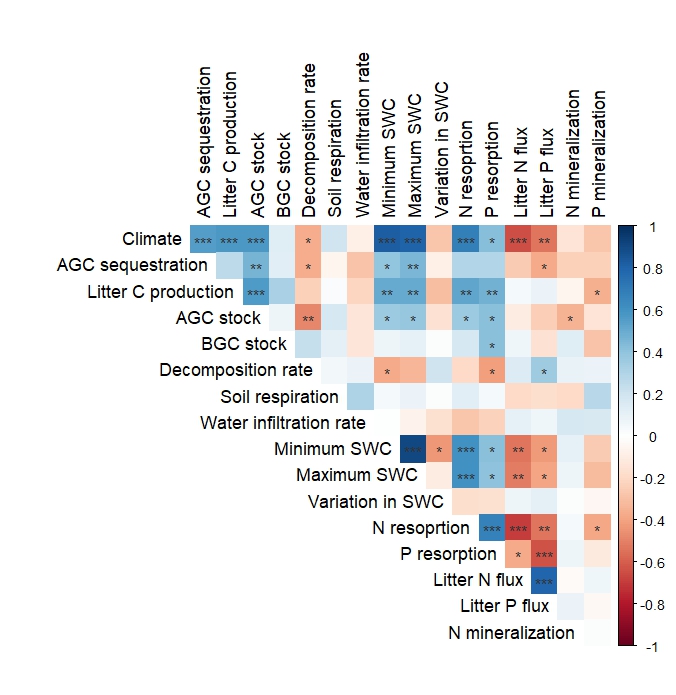


Figure S5. Results of the correlation analyses with the variables of 16 different ecosystem functions: aboveground carbon sequestration (AGC sequestration, ton ha^-1^ year^-1^), litter carbon production (Litter C production, ton ha^-1^ year^-1^), aboveground carbon stock (AGC stock, ton ha^-1^), belowground carbon stock (BGC stock, ton ha^-1^), litter decomposition rate (g day^-1^), soil respiration rate (µmol m^-2^ s^-1^), soil water infiltration rate (mm h^-1^), minimum soil water content (Minimum SWC, %), maximum soil water content (Maximum SWC, %), intra-annual variation in soil water content (Variation in SWC, %), nitrogen resorption rate (N resorption, %), phosphorus resorption rate (P resorption, %), litter nitrogen flux (Litter N flux, ton ha^-1^ year^-1^), litter phosphorus flux (Litter P flux, ton ha^-1^ year^-1^), mineralization rate of ammonium and nitrate (N mineralization, mg kg^-1^ day^-1^), and mineralization rate of phosphate (P mineralization, mg kg^-1^ day^-1^). Pearson pairwise correlation coefficients are given (* P < 0.05; ** P < 0.01, *** P < 0.001).

**REFERENCES**

Addo-Fordjour, P., & Rahmad, Z. B. (2013). Mixed Species Allometric Models for Estimating above-Ground Liana Biomass in Tropical Primary and Secondary Forests, Ghana. *International Scholalry Research Notices*, *2013*, 1–9. https://doi.org/10.1155/2013/153587

Aghimien, E. V., Osikabor, B., Adedeji, M. S., & Adams, O. T. (2020). Volume techniques for estimating standing and lying dead wood in Okomu national park, Edo state, Nigeria. *Biometrics & Biostatistics International Journal*, *9*(3), 111–116. https://doi.org/10.15406/bbij.2020.09.00308

Becker, G. S., Braun, D., Gliniars, R., & Dalitz, H. (2012). Relations between wood variables and how they relate to tree size variables of tropical African tree species. *Trees - Structure and Function*, *26*(4), 1101–1112. https://doi.org/10.1007/s00468-012-0687-6

Chao, K. J., Chen, Y. S., Song, G. Z. M., Chang, Y. M., Sheue, C. R., Phillips, O. L., & Hsieh, C. F. (2017). Carbon concentration declines with decay class in tropical forest woody debris. *Forest Ecology and Management*, *391*, 75–85. https://doi.org/10.1016/j.foreco.2017.01.020

Djagbletey, G. D., Adu-Bredu, S., DUah-Gyamfi, A., Aabeyir, R., Djagbletey, E. D., Akpalu, S. E., Adeyiga, G. K., Addo-Danso, S. D., Hagan Brown, W., Dabo, J., & Amponsah-Manu, E. (2020). *Wood Density Handbook for some West African Trees*.

Feng, C., Wang, Z., Ma, Y., Fu, S., & Chen, H. Y. H. (2019). Increased litterfall contributes to carbon and nitrogen accumulation following cessation of anthropogenic disturbances in degraded forests. *Forest Ecology and Management*, *432*(October 2018), 832–839. https://doi.org/10.1016/j.foreco.2018.10.025

Freschet, G. T., Pagès, L., Iversen, C. M., Comas, L. H., Rewald, B., Roumet, C., Klimešová, J., Zadworny, M., Poorter, H., Postma, J. A., Adams, T. S., Bagniewska-Zadworna, A., Bengough, A. G., Blancaflor, E. B., Brunner, I., Cornelissen, J. H. C., Garnier, E., Gessler, A., Hobbie, S. E., … McCormack, M. L. (2021). A starting guide to root ecology: strengthening ecological concepts and standardising root classification, sampling, processing and trait measurements. *New Phytologist*, *232*(3), 973–1122. https://doi.org/10.1111/nph.17572

Göransson, H., Welc, M., Bünemann, E. K., Christl, I., & Venterink, H. O. (2016). Nitrogen and phosphorus availability at early stages of soil development in the Damma glacier forefield, Switzerland; implications for establishment of N2-fixing plants. *Plant and Soil*, *404*(1–2), 251–261. https://doi.org/10.1007/s11104-016-2821-5

Hossain, M. A., Anik, A. R., Chakma, N., Johnson, K., Henry, M., Jalal, R., Carrillo, O., Scott, C., Birigazzi, L., Akhter, M., & Iqbal, Z. (2019). Estimation Procedures of Indicators and Variables of the Bangladesh Forest Inventory. *Forest Department and Food and Agriculture Organization of the United Nations, Dhaka, Bangladesh*.

Huaraca Huasco, W., Riutta, T., Girardin, C. A. J., Hancco Pacha, F., Puma Vilca, B. L., Moore, S., Rifai, S. W., del Aguila-Pasquel, J., Araujo Murakami, A., Freitag, R., Morel, A. C., Demissie, S., Doughty, C. E., Oliveras, I., Galiano Cabrera, D. F., Durand Baca, L., Farfán Amézquita, F., Silva Espejo, J. E., da Costa, A. C. L., … Malhi, Y. (2021). Fine root dynamics across pantropical rainforest ecosystems. *Global Change Biology*, *27*(15), 3657–3680. https://doi.org/10.1111/gcb.15677

Jongen, R., Hannula, S. E., De Long, J. R., Heinen, R., Huberty, M., Steinauer, K., & Bezemer, T. M. (2021). Plant community legacy effects on nutrient cycling, fungal decomposer communities and decomposition in a temperate grassland. *Soil Biology and Biochemistry*, *163*. https://doi.org/10.1016/j.soilbio.2021.108450

Matsuo, T. (2024). *Drivers and mechanisms of tropical secondary forest succession* [PhD thesis]. Wageningen University.

Nelson, D. A., & Sommers, L. (1983). Total carbon, organic carbon, and organic matter. *Methods of Soil Analysis: Part 2 Chemical and Microbiological Properties*, *9*, 539–579.

Novozamsky, I., Houba, V. J. G., van Eck, R., & van Vark, W. (1983). A novel digestion technique for multi-element plant analysis. *Communications in Soil Science and Plant Analysis*, *14*(3), 239–248. https://doi.org/10.1080/00103628309367359

Pérez-Harguindeguy, N., Díaz, S., Garnier, E., Lavorel, S., Poorter, H., Jaureguiberry, P., Bret-Harte, M. S., Cornwell, W. K., Craine, J. M., Gurvich, D. E., Urcelay, C., Veneklaas, E. J., Reich, P. B., Poorter, L., Wright, I. J., Ray, P., Enrico, L., Pausas, J. G., De Vos, A. C., … Cornelissen, J. H. C. (2013). New handbook for standardised measurement of plant functional traits worldwide. *Australian Journal of Botany*, *61*(3), 167–234. https://doi.org/10.1071/BT12225

Puletti, N., Canullo, R., Mattioli, W., Gawryś, R., Corona, P., & Czerepko, J. (2019). A dataset of forest volume deadwood estimates for Europe. *Annals of Forest Science*, *76*(3), 1–8. https://doi.org/10.1007/s13595-019-0832-0

Rayment, M. B., & Jarvis, P. G. (1997). An improved open chamber system for measuring soil CO2 effluxes in the field. *Journal of Geophysical Research Atmospheres*, *102*(D24), 28779–28784. https://doi.org/10.1029/97JD01103

van der Sande, M. T., Powers, J. S., Kuyper, T. W., Norden, N., Salgado-Negret, B., Silva De Almeida, J., Bongers, F., Delgado, D., Dent, D. H., Derroire, G., Do Espirito Santo, M. M., Dupuy, J. M., Fernandes, G. W., Finegan, B., Gavito, M. E., Hernández-Stefanoni, J. L., Jakovac, C. C., Jones, I. L., Das Dores Magalhães Veloso, M., … Poorter, L. (2022). Soil resistance and recovery during neotropical forest succession. *Philosophical Transactions of the Royal Society B: Biological Sciences*, *378*(1867). https://doi.org/10.1098/rstb.2021.0074
